# NaCl and urea modulate CD8^+^ T cell survival, renal accumulation, and response to BK virus

**DOI:** 10.1172/jci.insight.194570

**Published:** 2025-08-26

**Authors:** Peyman Falahat, Adrian Goldspink, Lucia Oehler, Jessica Schmitz, Julia Miranda, Islem Gammoudi, Jan Hinrich Bräsen, Niklas Klümper, Olena Babyak, Christian Kurts, Herrmann Haller, Marieta Toma, Sibylle von Vietinghoff

**Affiliations:** 1 Nephrology Section, Medical Clinic I, University Hospital Bonn, Rheinische Friedrich-Wilhelms Universität, Bonn, Germany.; 2 Division of Nephrology and Hypertension, Department of Internal Medicine, and; 3 Nephropathology Unit, Institute for Pathology, Hannover Medical School, Hannover, Germany.; 4 Hepatology Section, Medical Clinic I, University Hospital Bonn;; 5 Department of Urology;; 6 Institute of Molecular Medicine and Experimental Immunology, University Hospital Bonn; and; 7 Institute for Pathology, University Hospital Bonn, Rheinische Friedrich-Wilhelms Universität, Bonn, Germany.

**Keywords:** Immunology, Nephrology, Adaptive immunity, Epithelial transport of ions and water, Organ transplantation

## Abstract

BK virus nephropathy is a severe, graft-threatening complication of kidney transplantation that requires an effective T cell response. It typically emerges in the kidney medulla. Elevated osmolyte concentrations that dynamically respond to loop diuretic therapy characterize this environment. Here, BK viremia development in kidney graft recipients negatively correlated with loop diuretic therapy. The association remained significant in multivariable and propensity score–matched analyses. Kidney function was better preserved and CD8^+^ T cell abundance higher in loop diuretic–exposed allografts. CD8^+^ T cell densities in healthy human and murine kidney medulla were lower than in cortex and increased upon loop diuretic therapy in mice. As a potential underlying mechanism, kidney medullary NaCl and urea concentrations decreased primary human CD8^+^ T cell numbers in vitro by induction of cell death and limitation of proliferation, respectively. Both osmolytes downregulated interferon-related gene expression. NaCl induced p53-dependent apoptosis and upregulated Na^+^-transporter *SLC38A2*, which promoted caspase-3 activation. Both decreased T cell response and cytokine secretion in response to viral peptide and allogeneic tubular epithelial cell killing, components of anti-BK virus response in the kidney allograft. Our results propose osmolyte-mediated mitigation of CD8^+^ T cell function as a what we believe to be novel mechanism that impairs immune response to BK virus, the therapeutic potential of which is testable.

## Abstract 2

Our investigations demonstrate that high salt concentrations present in the inner kidney impair T-lymphocyte immune response to BK Virus, a pathogen that damages kidney grafts.

## Graphical abstract

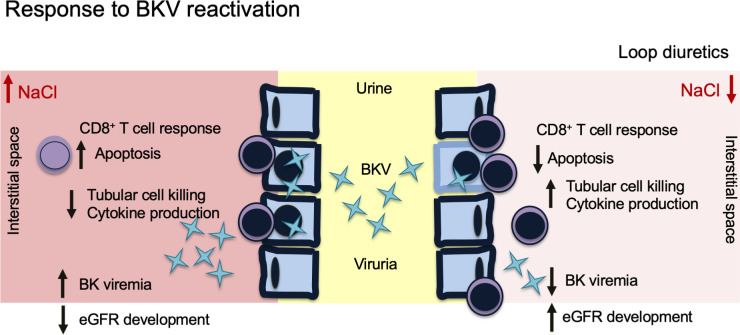

## Introduction

BK polyomavirus nephropathy (BKVn) is a typical complication of kidney transplantation (Ktx). The majority of the population is infected with this double-stranded DNA virus that can reactivate in urothelium and renal tubular epithelium, especially during immunosuppression (1, 2). After Ktx, BKVn affects up to 10% of recipients, most commonly during the first year after transplantation, and threatens graft survival (1, 2). BKVn manifests as tubulointerstitial nephritis with predilection for the kidney medulla (3, 4). BK viremia is observed in approximately 15%–26% of kidney graft recipients (5–7) and frequently indicates clinically relevant BKV reactivation (5, 8). BK viruria is even more common and usually precedes viremia and BKVn (5–7). However, BK viruria by itself does not necessarily associate with measurable kidney damage, possibly indicating a sufficient local immune response. Among BKVn risk factors (1), male recipient sex, tacrolimus as a calcineurin inhibitor, rejections, and a deceased donor associated with viremia in a meta-analysis (6). Current therapeutic options mainly consist of immunosuppression reduction, a switch to mTOR inhibitors, and high-dose intravenous immunoglobulins (8–10). They carry a high risk of graft rejection. Novel therapeutic strategies are needed.

Antiviral immune mechanisms need to target BK virus (BKV) in the complex environment of an immunosuppressed host with a non-HLA-identical kidney. In the absence of a suitable animal model (11), pathophysiologic understanding is based on in vitro assays and ex vivo correlations with clinical outcome. While other arms of the adaptive immune system, namely a neutralizing anti-BKV immunoglobulin titer (7) and CD4^+^ helper T cells (12, 13), contribute, antiviral responses chiefly rely on antigen presentation by the tubular epithelium and urothelium via MHCI to cytotoxic CD8^+^ T cells (14, 15). BKV-specific cytotoxic CD8^+^ T cells can be detected in the blood of healthy individuals (16), mainly with an effector-memory phenotype and able to produce multiple cytokines, such as interleukin-2 (IL-2), tumor necrosis factor-α (TNF-α), and interferon-γ (IFN-γ). Circulating effector memory CD8^+^ T cells decreased during BKV reactivation after Ktx (17). Conversely, in another investigation, more cytokine-producing effector memory CD8^+^ T cells circulated in blood of patients who rapidly cleared BKV (18). This suggests a substantial systemic CD8^+^ T cell contribution to anti-BKV response, requiring successful effector function after recruitment to the kidney medulla.

Host response in the kidney medulla occurs in an environment that differs markedly from other bodily compartments, namely by high osmolarity (19). NaCl and urea are the main mobile osmolytes in the human kidney. Their gradient dynamically responds to volume and electrolyte requirements. Pharmacologically, it is frequently modulated by loop diuretics, which rapidly deplete sodium from the kidney medulla (20, 21). Recent research has started to elucidate how elevated NaCl concentrations modulate kidney regeneration (22) and functions of renal nonimmune, innate, and adaptive immune cells in general. Kidney tubular epithelium responds with chemokine production to medullary NaCl concentrations (23–25), while urea was inert or even suppressed their production (25). Data on myeloid cells are mixed, with some results suggesting improvement of bactericidal function (23). Others rather found M2-type, reparative macrophage differentiation in the presence of increased NaCl (24, 26). NaCl also enhanced programmed myeloid cell death (27), similar to an observation in corneal dendritic cells (28). Dendritic cell–mediated CD8^+^ T cell activation was reduced in a kidney medullary NaCl environment (29). Regarding adaptive immune function, namely the cellular immune response, extensive data on CD4^+^ T cells show enhanced T_H_17 cell differentiation (30), especially in inflammatory environments. This may also apply to T_H_1 cells (31), while T_REG_ cell function was rather impaired (32). CD8^+^ cytotoxic T cells remain incompletely explored regarding their response to ambient NaCl. A recent publication shows a role for osmosensor NFAT5 in CD8^+^ T cell exhaustion during antitumor response (33). Two others instead propose an immunostimulatory function of NaCl in naive CD8^+^ T cells (34, 35). Effects on renal adaptive immunity have not been reported to our knowledge.

Starting from our results that NaCl promotes and loop diuretic therapy mitigates myeloid cell recruitment into the kidney and that NaCl induces their programmed cell death and overall improves renal antibacterial host response (24, 25, 27), we here investigated the situation in BKV reactivation, a condition requiring adaptive CD8^+^ T cell response. Clinical data indicated improved host response during loop diuretic therapy. Mechanistic investigations in CD8^+^ T cell response to kidney osmolytes were performed.

## Results

### Loop diuretic therapy negatively associates with BK viremia in renal transplant recipients.

**Figure 1:**
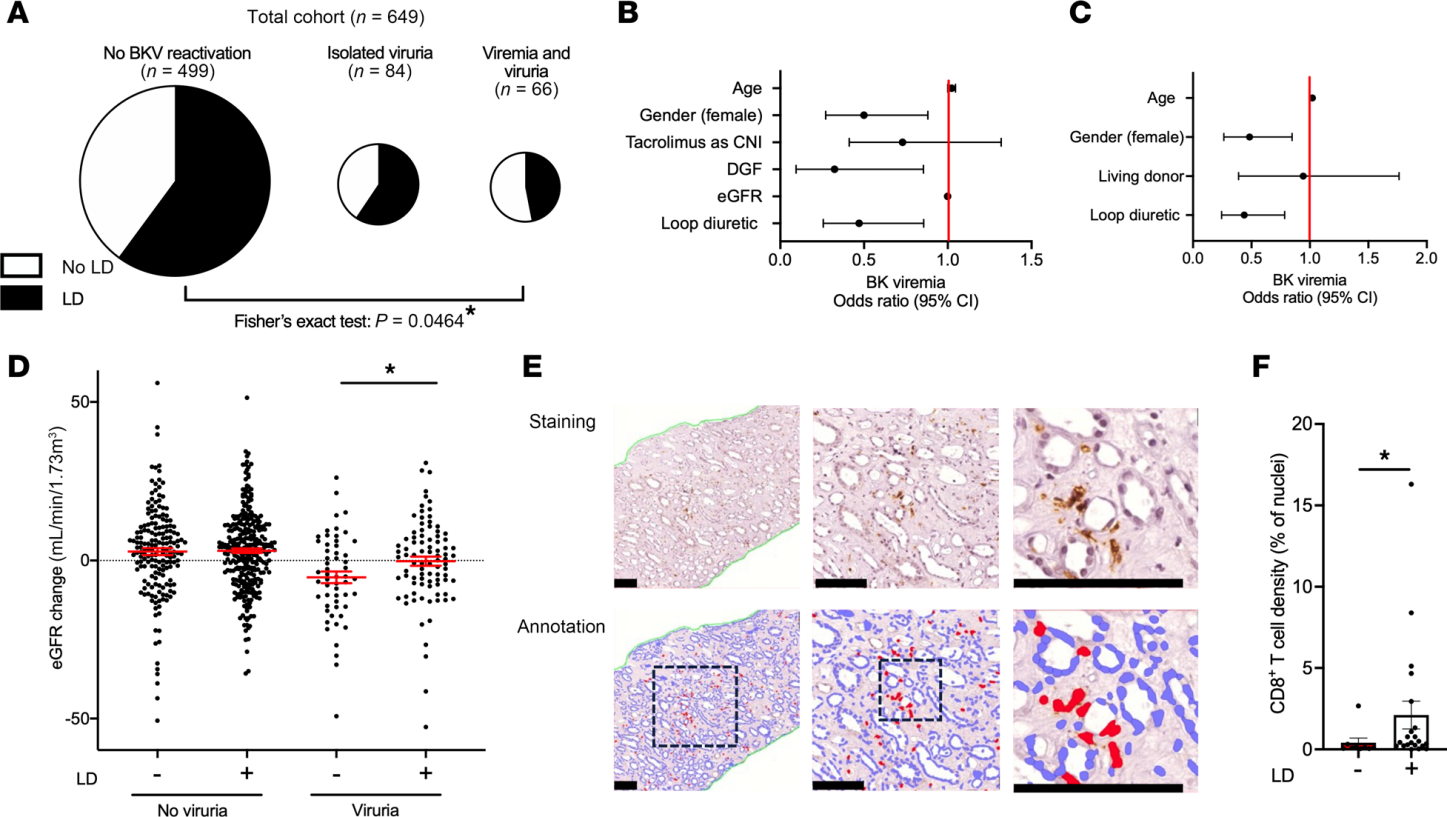
BKV reactivation, outcome, and renal CD8^+^ T cell accumulation in kidney transplant recipients with and without loop diuretic therapy. (**A**) Proportion of loop diuretic–treated (LD-treated) patients among BKV-negative (199 without, 300 with LD); viruric, but not viremic (27 without, 57 with LD); and viremic (35 without, 31 with LD) patients (Fisher’s exact test). (**B** and **C**) Multiple logistic regression odds ratios with 95% confidence interval of BK viremia development of major variables in univariable regression, including age (**B**: 1.02 [1.0–1.05], **C**: 1.02 [1.0–1.05]), sex (**B**: 0.5 [0.27–0.88], **C**: 0.48 [0.26–0.85]), type of calcineurin inhibitor (CNI, 0.72 [0.41–1.32]), delayed graft function (DGF, 0.32 [0.09–0.85]), estimated glomerular filtration rate (eGFR, CKD-EPI, 1.0 [0.98–1.02]), donor type (0.95 [0.39–1.76]), and LD use (**B**: 0.47 [0.25–0.86], **C**: 0.44 [0.24–0.78]). (**D**) eGFR development during the first year after Ktx in patients with and without BK viruria and LD therapy (Mann-Whitney test). (**E** and **F**) Cytotoxic CD8^+^ T cell densities of 3-month postimplantation kidney allograft biopsies were quantified as proportion of all nuclei. Computer-assisted annotations are shown in the lower panel (**E**, bars indicate 100 μm). Patients were stratified according to LD use (**F**, statistical analysis by Mann-Whitney test). *P < 0.05.

We analyzed the relation of BK viremia to established risk factors and loop diuretic use in kidney allograft recipients during the chief BKV reactivation period, the first year after Ktx (Table 1) (5, 6, 8, 36). Among the 649 patients transplanted 2008–2017 at Hannover Medical School, Germany, who participated in at least 12 months of urine and blood BKV screening by PCR and follow-up at our institution as detailed in Methods, 23% developed viruria and 10% viremia. Loop diuretic–treated patients were significantly underrepresented among the group who developed viremia (Figure 1A). BK viremia significantly negatively associated with loop diuretic therapy (*r* = –0.09, *P* = 0.025) and its dose (*r* = –0.1, *P* = 0.007, Spearman’s).

The relation of BK viremia to this and other, previously established clinical risk factors and characteristics that differed significantly between patients receiving loop diuretic was analyzed in more detail. Table 2 shows the results of regression analysis of known BKV reactivation risk factors, including graft rejection. There were significant negative correlations of BK viremia with female sex, delayed graft function (DGF), and loop diuretic use. These and the other factors with a *P* < 0.3, namely age, use of tacrolimus as calcineurin inhibitor (CNI), and eGFR, were tested in a multivariable logistic regression analysis. Resulting odds ratios and 95% confidence intervals are shown in Figure 1B. Sex, DGF, and loop diuretic therapy remained significant. Given that a living donor is a major predictor of eGFR, DGF, cold ischemia time, and ABO blood group incompatibility, all of which differed in incidence between loop diuretic recipients and other patients (Table 1), we also recalculated odds ratios after replacing these by the factor “living donor.” Also in this model, female sex and loop diuretic use were significant protective factors against BK viremia (Figure 1C). After propensity score matching for age at transplantation, recipient sex, cold ischemia time, living versus deceased donor type, HLA mismatches, ABO incompatibility, CNI use, tacrolimus use, mTOR inhibitor use, baseline eGFR, DGF, and occurrence of rejection (Methods), the odds ratio to develop BK viremia also was significantly lower in the loop diuretic group (OR = 0.44, 95% CI [0.20, 0.91], *P* = 0.030).

These data propose a to the best of our knowledge novel protective association of loop diuretic therapy with BK viremia after Ktx.

### Less loss of eGFR upon BK viruria during loop diuretic therapy.

We next addressed renal outcome after BKV reactivation. Viral loads in plasma, urine, and biopsy results did not differ significantly between the loop diuretic–treated and untreated groups (Supplemental Table 1; supplemental material available online with this article; https://doi.org/10.1172/jci.insight.194570DS1). Therapeutic measures taken in viremic patients followed local standards and included mycophenolate dose reduction, switch from mycophenolate to an mTOR inhibitor, CNI cessation, and high-dose intravenous immunoglobulin in this order. They were similarly applied in both groups (Supplemental Table 1). There was no recorded graft loss due to BKVn in either group. Patients receiving loop diuretic nonsignificantly trended to be biopsied and switched from mycophenolate to mTOR inhibitor more often, possibly because of a lower starting eGFR already before viremia onset. Long-term eGFR development was nominally but nonsignificantly more stable in the loop diuretic–treated group (Supplemental Figure 1).

Urinary viral shedding was universally detectable before viremia in our cohort. It may be considered a milder stage of locally controlled BKV reactivation (1, 5, 6). Kidney functional development in the viruric group receiving loop diuretic was significantly better than in viruric patients without loop diuretic (Figure 1D and Supplemental Table 2). In the absence of BKV reactivation, eGFR development did not differ between loop diuretic–treated and nontreated patients. The baseline eGFR values of the loop diuretic recipients with and without subsequent viruria did not differ (37 ± 15 vs. 39 ± 15 mL/min/1.73 m², *P* = 0.55). Similar effects were seen in males and females (Supplemental Figure 2).

The protective associations of loop diuretic use with viremia development and eGFR loss during viruria are consistent. They may imply a pathophysiologic relevance of loop diuretic effects for renal host response during viral reactivation.

### Increased CD8^+^ T cell frequency in human kidney grafts with loop diuretic therapy.

As antiviral responses rely on antigen presentation to lymphocytes, namely cytotoxic CD8^+^ T cells (14, 15), we analyzed renal T cell subpopulations in grafts with and without loop diuretic therapy. Immunostainings and computer-assisted quantification were conducted in all available 3 months posttransplantation biopsies from a 2-year subcohort (staining examples and tissue areas in Supplemental Figure 3). A total of 65 patients’ tissues were assessed, chiefly surveillance biopsies at a mean of 92.9 ± 2.6 days after Ktx. Time after Ktx and Banff classifications did not significantly differ between patients receiving loop diuretic and not (Supplemental Tables 3 and 4). Significantly higher CD8^+^ cytotoxic T cell densities compared with total cell numbers were found in loop diuretic–treated patients’ medulla (Figure 1, E and F, and Supplemental Table 5). In contrast, medullary pan–T cell marker CD3 and T_H_ cell marker CD4 densities decreased with loop diuretic use (Supplemental Figure 4). Professional antigen-presenting cells expressing DC-SIGN (CD209^+^) were unaffected by loop diuretic therapy (Supplemental Figure 5). Six T cell– and 2 antibody-mediated rejections and 1 case of BKVn were diagnosed, with 8 cases classified as borderline. The principal observation of higher medullary CD8^+^ T cell and decreased CD4^+^ T cell densities during loop diuretic therapy remained similarly apparent if these were excluded (Supplemental Figure 6). It also persisted for females and males, and deceased and living donor kidneys, despite marked variations in absolute values (Supplemental Figure 7).

These results suggest that CD8^+^ T cells’ response to renal osmolytes should be investigated in vivo and in vitro.

### Lower CD8^+^ T cell densities in human and murine kidney medulla than cortex.

**Figure 2:**
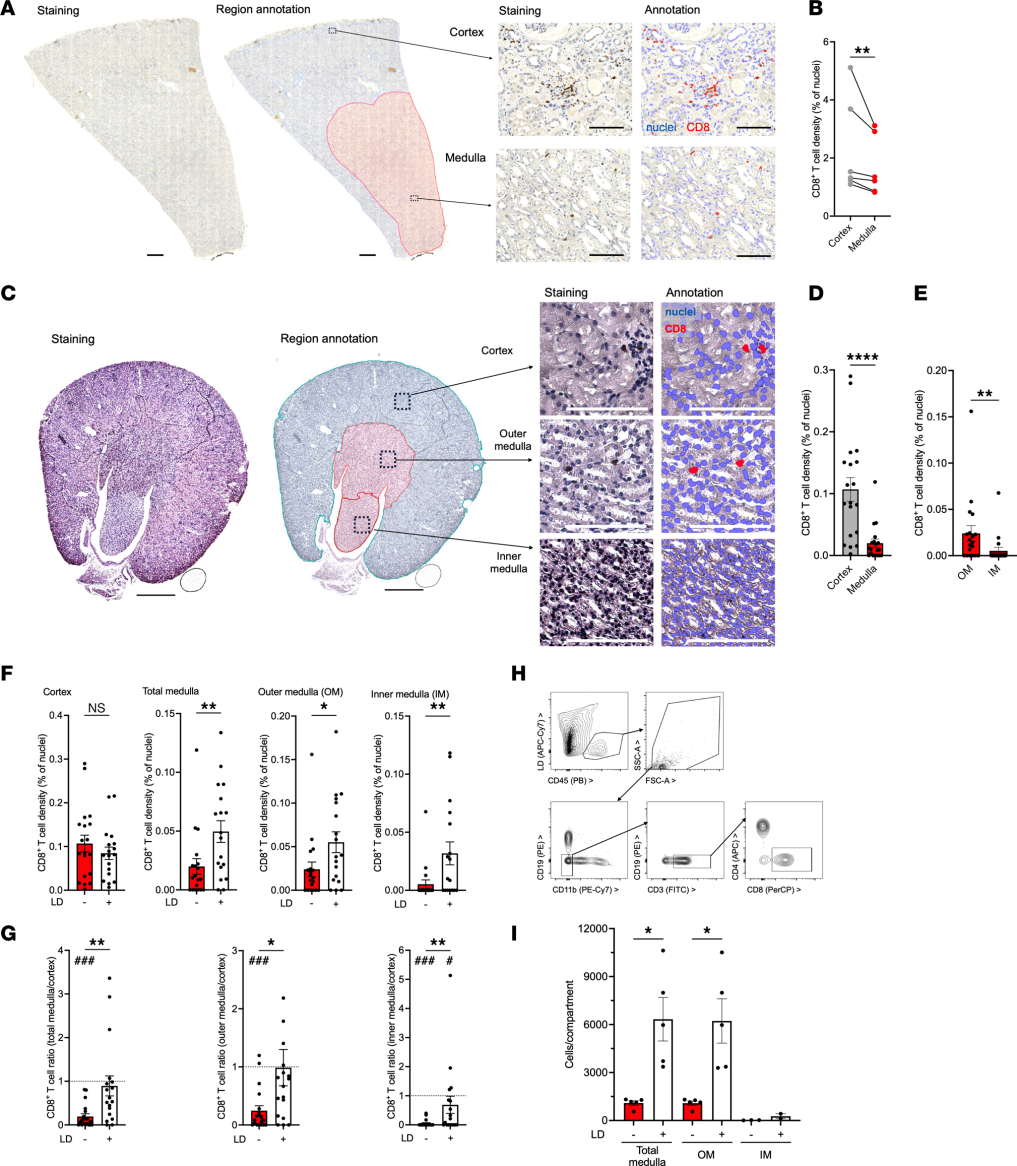
CD8^+^ T cell distribution in human and murine kidney compartments. (**A** and **B**) CD8^+^ T cell densities were quantified after immunostaining and computer-assisted annotation in cortex and medulla of healthy human kidney tissues (example of a whole-slide scan, region annotation, nuclear and CD8 staining, and annotation in **A**; bars indicate 1 mm and 100 μm, respectively; **B**, ratio-paired Student’s *t* test, *n* = 6 donors). (**C**–**G**) CD8^+^ T cell densities were assessed in murine kidney cortex and outer (OM) and inner (IM) medulla by histology (examples of staining and annotation in **C**; bars indicate 1 mm and 100 μm; **D** and **E**: statistical analysis of *n* = 19 mice, Mann-Whitney tests). (**F** and **G**) CD8^+^ T cell densities in the indicated compartments (**F**) and their ratios to the cortex (**G**) were compared with kidneys from LD furosemide–pretreated mice (*n* = 18). Mann-Whitney tests LD versus non-LD (significant *P* values indicated by *). Dashed lines indicate equal density in cortex and medulla (Wilcoxon test against 1, significant *P* values indicated by ^#^). (**H** and **I**) CD8^+^ T cells were enumerated by flow cytometry in total OM and IM from mice with and without LD pretreatment (**H**, flow cytometric gating strategy; **I**, statistical analysis of *n* = 5 per group, Student’s *t* tests with Welch’s correction by Kruskal-Wallis test). *P < 0.05, **P < 0.01, ***P < 0.001, ****P < 0.0001. ^#^*P* < 0.05, ^###^*P* < 0.001.

To test for a relation of the kidney osmotic gradient and CD8^+^ T cell densities, we first assessed healthy human kidney cortex and medulla in patients without loop diuretic therapy. In all donors, cortical CD8^+^ T cell density was higher than medullary (Figure 2, A and B). In mice, a similar gradient was observed (Figure 2, C and D). In addition, in murine histology allowing reliable distinction, the inner medulla contained even lower counts than the outer medulla (Figure 2E). Next, mice were treated with loop diuretic. This significantly increased medullary CD8^+^ T densities by both histology and flow cytometry. The gradient between the compartments disappeared (Figure 2, F–I).

The observed dynamic response to loop diuretic therapy in healthy kidney is consistent with CD8^+^ T cell modulation by environmental osmolytes.

### Renal osmolytes NaCl and urea modulate CD8^+^ T cell proliferation and survival.

**Figure 3:**
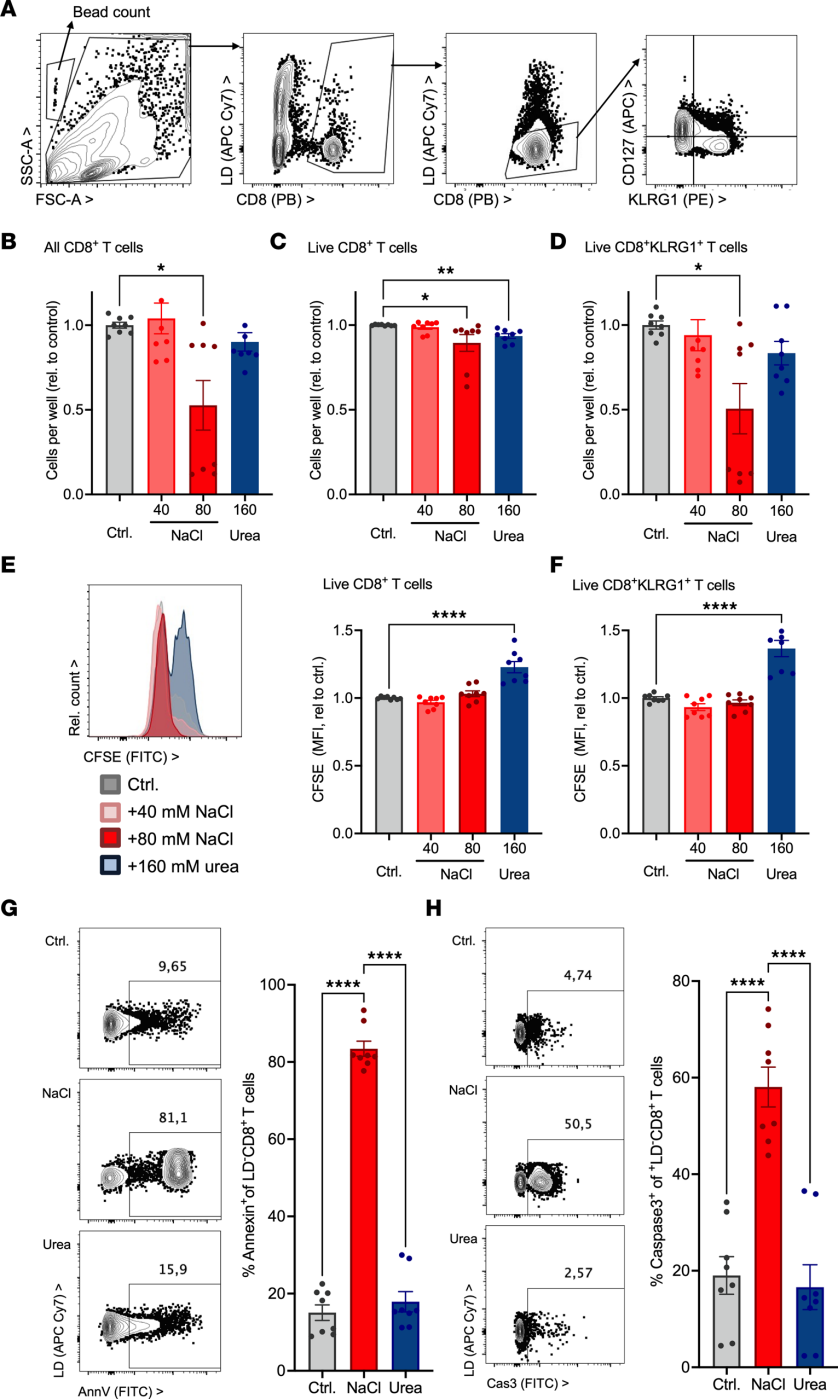
Kidney medullary level of NaCl and urea modulates T cell proliferation and survival. (**A**–**H**) Human adhesion-depleted PBMCs were cultured with allogeneic stimulation by HK2 human tubular epithelial cells with and without addition of NaCl and urea in the indicated concentrations (baseline NaCl 144.4 mM, 312 mOsm total osmolarity, final NaCl 184.4 and 224.4 mM, final osmolarities 392 und 472 mOsm). (**A**–**D**) Numbers of all CD8^+^ T cells (**B**), live CD8^+^ T cells (**C**), and live CD8^+^KLRG1^+^ T cells (**D**) were enumerated by flow cytometry on day 3 (gating in **A**, *n* = 8 from 4 donors in 2 independent experiments, Dunn’s). (**E** and **F**) PBMCs were labeled with CFSE before start of the experiment, and its dilution in live CD8^+^ T cells (**E**) and live CD8^+^KLRG1^+^ T cells (**F**) was assessed on day 3 (flow cytometric example, *n* = 8 from 4 donors in 2 independent experiments, Dunnett’s after ANOVA). (**G** and **H**) Apoptosis markers annexin V binding (**G**) and caspase-3 (**H**) were assessed by flow cytometry after 20 hours of culture with and without additional 80 mM NaCl or equimolar urea (160 mM, examples and *n* = 8 from 4 donors in 2 independent experiments, Dunnett’s). *P < 0.05, **P < 0.01, ****P < 0.0001.

To address possible underlying factors of the renal CD8^+^ T cell gradient, proliferation and cell death modulation were investigated. To this end, human primary CD8^+^ T cell cultures with an alloimmune stimulus, HK2 renal tubular epithelial cells, were performed in the presence of elevated NaCl and urea in concentrations and total osmolarity comparable to the human kidney medulla (20, 37, 38). On day 3 of culture, significantly fewer total and live CD8^+^ T cells were recovered from cultures with additional 80 mM NaCl (224 mM total extracellular Na^+^, total osmolarity 472 mOsm) compared with control with added 40 mM NaCl or equimolar 160 mM urea (Figure 3, A–C). This phenotype was further aggravated at an additional 120 mM NaCl but not induced by mannitol tonicity control (Supplemental Figure 8A). HK2 cell phenotype was unaffected by additional 80 mM NaCl (Supplemental Figure 8B). The number of CD8^+^ T cells expressing the effector marker KLRG1 (15) was significantly lower (Figure 3D).

Proliferation and cell death were explored as potential underlying mechanisms. CD8^+^ T cell proliferation studied by CFSE dilution was inhibited by urea (Figure 3, E and F) but not NaCl. In contrast, NaCl, but not urea, dose-dependently induced CD8^+^ T cell death characterized by annexin V binding and caspase-3 cleavage, consistent with apoptosis (Figure 3, G and H). Neither sodium channel NCX inhibitors K-BR and SEA, Na^+^K^+^ATPase inhibitor digitoxin, calcium chelation, PAD4 inhibitor GSK484, nor gasdermin D inhibitor disulfiram changed overall cell survival (data not shown), arguing for a major upstream mediator.

These results demonstrate differential mechanisms for how NaCl and urea affect CD8^+^ T cell numbers.

### Differential regulation of CD8^+^ T cell gene expression by NaCl and urea.

**Figure 4:**
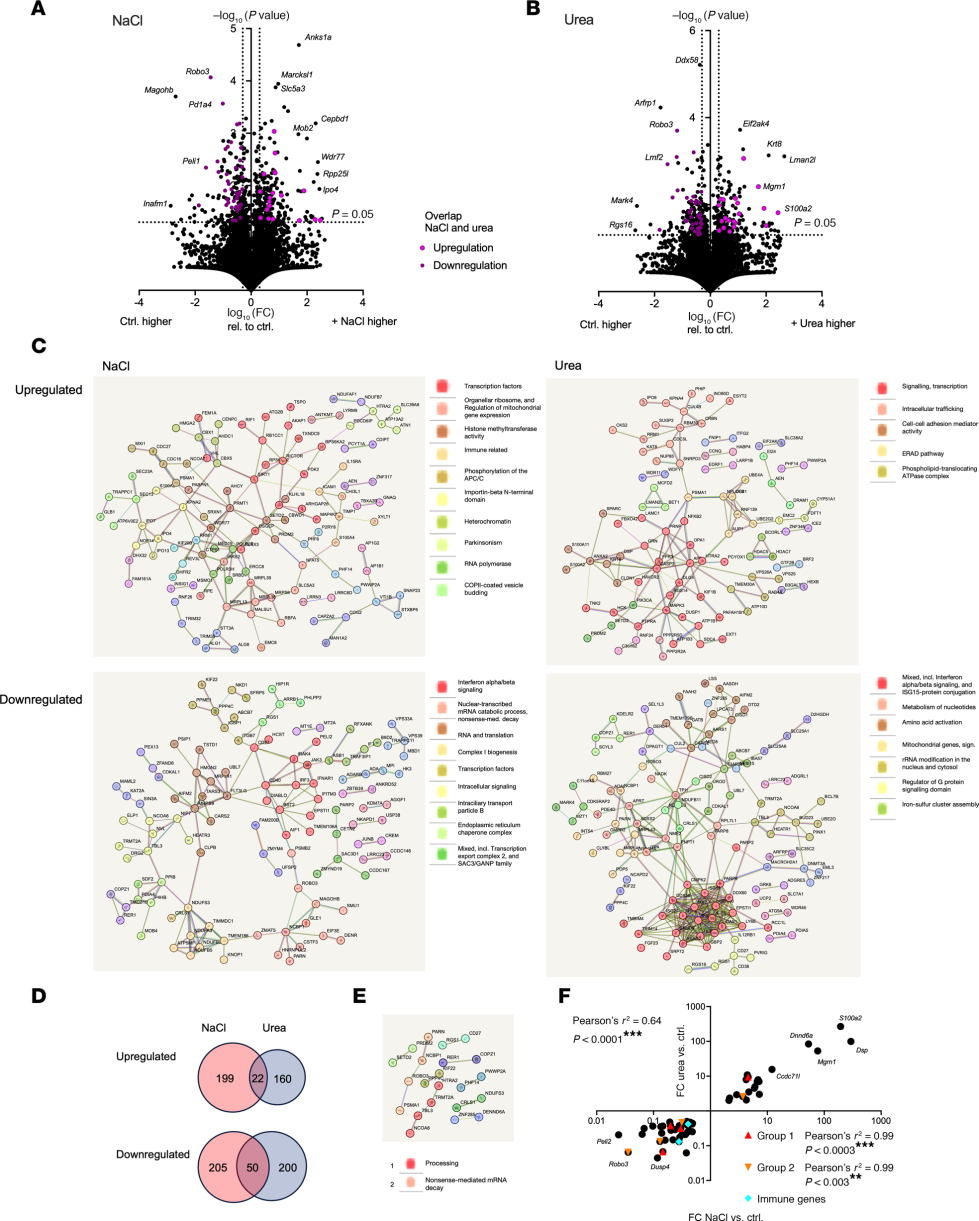
Differential modulation of human CD8^+^ T cell gene expression by NaCl and urea. (**A**–**F**) Human adhesion-depleted PBMCs were cultured with allogeneic stimulation by HK2 human tubular epithelial cells with and without addition of 80 mM NaCl or 160 mM urea for 72 hours. After 6 hours of allogeneic restimulation with fresh tubular epithelial cells, viable CD8^+^ T cells were isolated by flow cytometry and subjected to RNA sequencing (*n* = 4 donors). (**A** and **B**) Volcano plots of regulated genes in NaCl (**A**) and urea (**B**) conditions compared with control (dotted lines indicate a raw *P* < 0.05 and 2-fold increase or decrease). (**C**) Natural clustering analysis of gene groups regulated more than 2-fold up or down in either NaCl or urea condition was performed, and names of clusters with 5 or more genes are shown as proposed by String. (**D**) Venn diagram of parallel up- and downregulated genes for the NaCl and urea conditions (2-fold regulation, raw *P* < 0.05). (**E**) Natural clustering analysis of parallel regulated genes was performed by String, and clusters with 3 or more genes are shown as proposed by String. (**F**) Correlation of fold-change expression change of commonly regulated genes (*n* = 72). The subgroups defined by natural clustering (**E**) are shown separately.

To detect potentially mechanistically underlying alterations and to obtain a more complete view of gene regulation patterns exercised by NaCl and urea, RNA sequencing was performed. Viable CD8^+^ T cells were examined after 3 days’ culture and 6 hours’ restimulation (Supplemental Figure 9A). Very similar total read counts were obtained (Supplemental Figure 9B). A total of 12,352 genes were expressed in at least 1 control sample and included in the analysis (Figure 4). With additional NaCl, 189 genes were upregulated and 186 downregulated at least 2-fold and analyzed further (Figure 4A). With urea, such upregulation was observed for 148 and downregulation for 188 genes (Figure 4B). Investigation of functional gene groups revealed IFN-signaling genes as the most abundant group among downregulated genes in both NaCl and urea conditions (Figure 4C). However, the individual genes differed between them. In addition, mitochondrial and cellular transport molecules were downregulated by NaCl, and RNA modification and metabolic genes by urea. A number of interaction networks were discovered, and transcriptional and translational mediators were most prominent in the NaCl condition. Regarding common regulation in NaCl and urea, 22 genes were upregulated and 50 downregulated in both conditions (Figure 4D, also annotated in the volcano plots of Figure 4, A and B, and full gene lists in Supplemental Tables 6 and 7). No significantly contrarily regulated genes were observed. Small functional gene groups among the coregulated 72 genes affected translational regulation (Figure 4E). Extent of gene regulation by NaCl and urea was very similar, both overall and for the functional groups (Figure 4F).

In summary, NaCl and urea regulate multiple and diverse gene groups in CD8^+^ T cells, with a functional overlap in downregulation of IFN signaling and antiviral response.

### CD8^+^ T cell gene regulation by NaCl and urea corresponds to surface protein expression, renal regulation, and impact on cell death.

**Figure 5:**
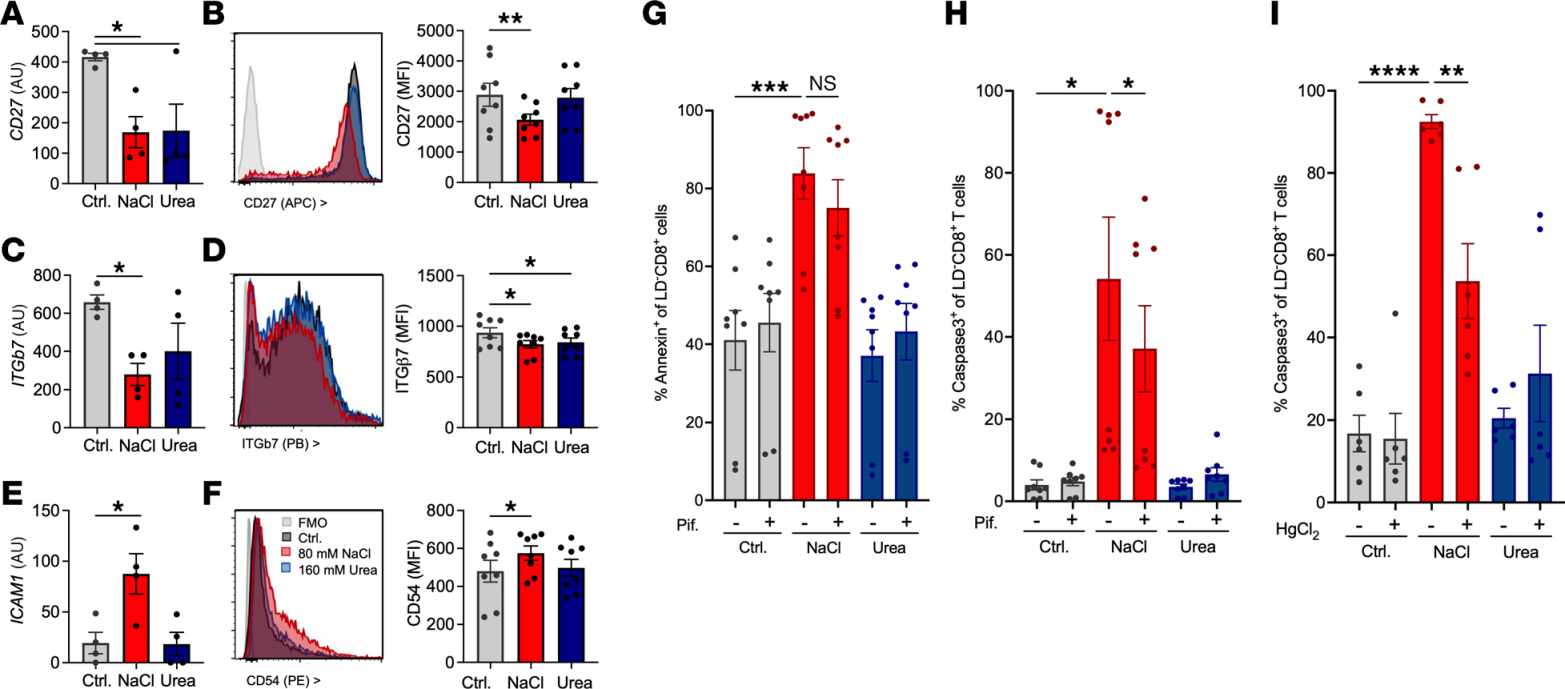
Regulation of surface protein expression and response of NaCl-induced cell death to p53 and SNAT2 pharmacologic inhibition. (**A**–**F**) Regulation of *CD27* (**A** and **B**), *ITGb7* (**C** and **D**), and *ICAM1* (**E** and **F**) mRNA assessed by RNA sequencing (**A**, **C**, and **E**, experimental setup in Figure 3, *n* = 4 donors, Dunnett’s after ANOVA) and surface expression on live CD8^+^CD3^+^CD11b^–^ T cells after overnight incubation with additional NaCl (80 mM) or urea (160 mM) (**B**, **D**, and **F**, *n* = 8 from 4 donors in 2 experiments, Dunnett’s after ANOVA). (**G** and **H**) Apoptosis markers annexin V binding (**G**) and caspase-3 (**H**) were assessed by flow cytometry after 20 hours culture with and without additional 80 mM NaCl or equimolar urea (160 mM) in the presence of p53 inhibitor pifithrin (30 μM) or DMSO solvent control (*n* = 8 from 4 donors in 2 independent experiments, Šídák’s after ANOVA). (**I**) Apoptosis marker caspase-3 was assessed by flow cytometry after 6 hours’ culture with and without additional 80 mM NaCl or equimolar urea (160 mM) in the presence of SNAT2 (gene name *SLC38A2*) blocker HgCl_2_ (10 μM, *n* = 6 from 3 donors in 2 independent experiments, Šídák’s after ANOVA). *P < 0.05, **P < 0.01, ***P < 0.001, ****P < 0.0001. Pif, pifithrin.

To investigate the relevance of gene for protein regulation, we quantified T cell surface proteins by flow cytometry. Downregulation of surface receptor CD27, an important factor in CD8^+^ T cell memory formation (39), and its interaction partner β7 integrin by NaCl was equally detected on the protein level (Figure 5, A–D). Adhesion molecule *ICAM1* (CD54) was upregulated by NaCl on both mRNA and protein levels (Figure 5, E and F), consistent with an impact on gene expression regulation for surface protein levels.

To test for renal relevance, we investigated genes regulated in parallel by NaCl and urea for their expressional ratio between total healthy medulla and cortex (Supplemental Figure 10). Extent of regulation aligned significantly for genes regulated by NaCl (*r*^2^ = 0.143, *P* = 0.0037) and urea (*r*^2^ = 0.116, *P* = 0.0087). Regulated genes were also analyzed in relation to data from naive antibody-stimulated CD8^+^ T cells (Supplemental Figure 11) (34, 35). Among the few parallel regulated genes with more than 2-fold change (Supplemental Table 8) were membrane transporter and channel subunits (*ATP1B3*, *SLC5A3*), amino acid uptake and metabolism (*PGPG*, *SLC5A3*, *CTPS1*), a few immune genes (*CD27*, *JAK3*, *LMBR1L*, downregulated), apoptosis protection (*NUAK2*, downregulated), histone methylation genes (*KDM7A*, *PRMT1*, *ASRGL1*, *AKAP1*), and mitochondrial genes (*MRPS6*, *RBFA*, both upregulated), as well as cell cycle inhibitor *CDK2AP1* (upregulated). Extent of regulation between upregulated genes was very similar (Supplemental Figure 11, C and D). However, NaCl-induced gene regulation in both datasets of naive antibody-stimulated CD8^+^ T cells failed to associate with the kidney (Supplemental Figure 11E). Urea upregulated CD8^+^ T cell exhaustion marker *HAVCR2* and NaCl *SLAMF6*, but other exhaustion markers described in tolerated murine kidney allografts (40) were unaltered (Supplemental Figure 12).

Cell death inducer *AEN* was upregulated, a finding also applicable to naive T cells (Supplemental Figure 13, A and B) (34, 35). *AEN* is regulated by tumor suppressor p53 (41). Indeed, p53 inhibition with pifithrin significantly improved cell survival in the presence of elevated NaCl and decreased annexin V binding and caspase-3 activation (Figure 5, G and H). In relation to the kidney gradient, among the most prominent coregulated gene was Na–amino acid co-transporter SNAT2 (gene name *SLC38A2*), which was equally regulated in naive T cells (Supplemental Figure 13, C and D) (34, 35). SNAT2 recently was implicated in T cell differentiation (42) and protection of kidney medullary epithelium from ferroptosis (43). Mercury is the most specific available pharmacologic blocker (44). While toxic upon long-term application, SNAT2 inhibition by mercury significantly inhibited caspase-3 cleavage after 6 hours in response to elevated NaCl (Figure 5I).

Impact on CD8^+^ T cell protein expression and cell death, together with parallel regulatory patterns in the kidney, suggest that osmolyte-induced gene expression changes may be relevant for CD8^+^ T cell function in the renal environment.

### Diminished allogeneic tubular cell killing and antiviral-peptide response by NaCl-exposed CD8^+^ T cells.

To start to address the mechanistic impact of NaCl and urea on T cell functions preventing BKVn, allogeneic epithelial cell killing and response to BKV peptide were studied.

**Figure 6:**
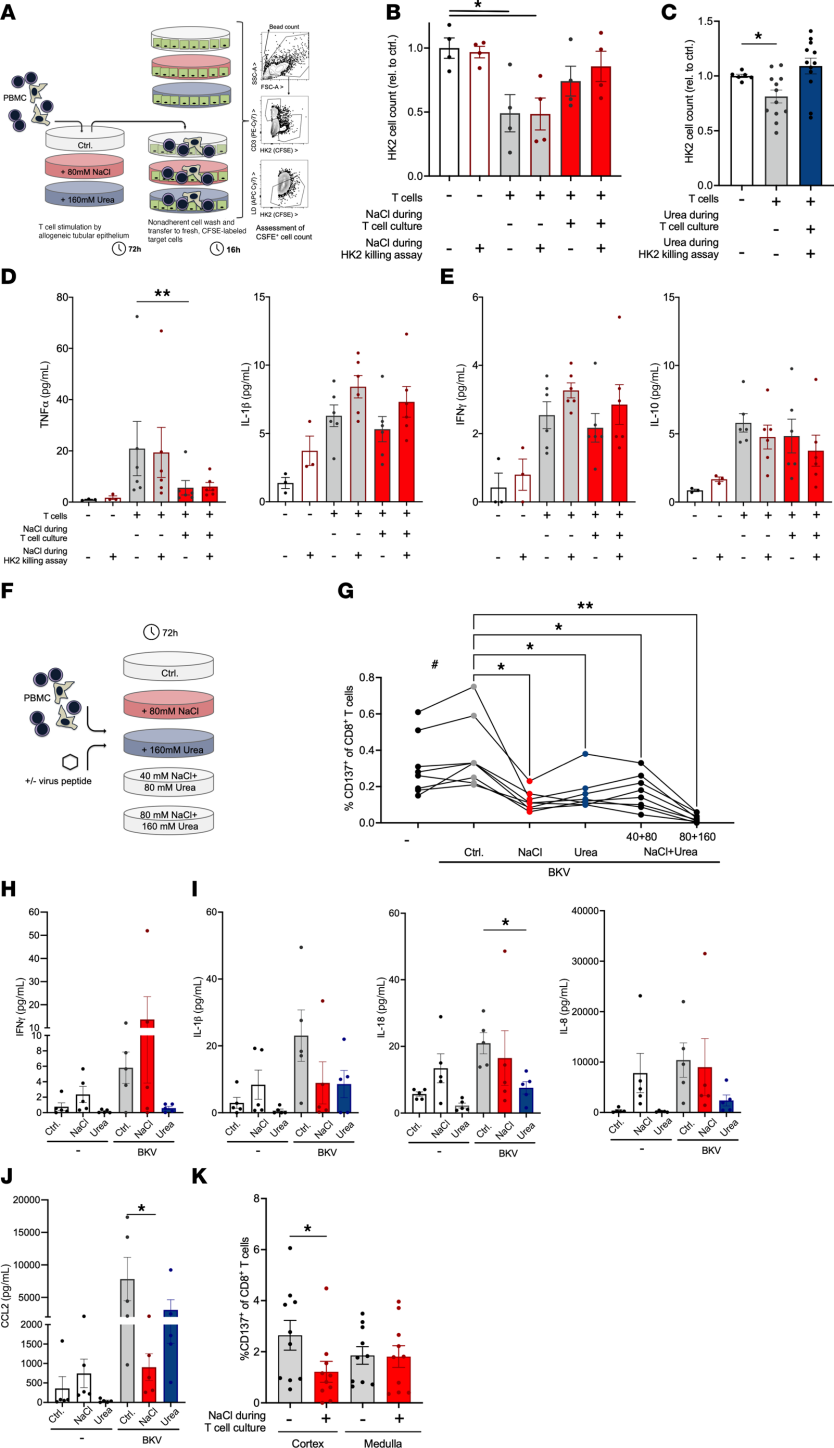
Response to BKV peptide and tubular epithelial cell killing are dampened by environmental NaCl and urea. (**A**–**E**) After 3 days’ culture with additional 80 mM NaCl, 160 mM urea, or control, adhesion-depleted PBMCs were added to CFSE-labeled allogeneic HK2 human tubular epithelial cells with and without addition of NaCl as outlined in **A**. (**B** and **C**) After 16 hours, live HK2 cell counts were assessed by flow cytometry for NaCl (**B**, *n* = 4 from 2 donors in 2 independent experiments) and urea (*n* = 12 from 6 donors in 3 independent experiments, Šídák’s after ANOVA). (**D** and **E**) After 3 days’ culture with additional 80 mM NaCl or control, equal numbers of magnetically enriched CD8^+^ T cells were added to fresh allogeneic HK2 human tubular epithelial cells with and without additional 80 mM NaCl. After 16 hours, supernatant TNF-α, IL-1β, IFN-γ, and IL-10 were assessed by cytometric bead assay (*n* = 6 T cell donors in 3 independent experiments, Dunn’s). (**F**–**J**) PBMCs were cultured with and without stimulation with BKV peptide in the absence or presence of additional 80 mM NaCl, equimolar 160 mM urea, their combinations or control for 3 days (experimental setup in **F**). (**G**) The proportion of responsive CD137^hi^CD8^+^ T cells was quantified by flow cytometry (gating in Supplemental Figure 16A) (statistical analysis of *n* = 8 from 4 donors in 2 independent experiments, ^#^paired Student’s *t* test of stimulation, *Dunnett’s after ANOVA of peptide conditions). Supernatant IFN-γ (**H**), IL-1β, IL-18, IL-8 (**I**), and CCL2 (**J**) were assessed by cytometric bead assay (*n* = 5 T cell donors in independent experiments, Dunn’s of peptide conditions). (**K**) In single-cell suspensions of human kidney cortex and medulla, proportion of responsive CD137^hi^ among CD8^+^ T cells was assessed after 3 days of stimulation with BKV peptide during culture in control and additional 80 mM NaCl conditions (gating in Supplemental Figure 16B; *n* = 6 experiments from *n* = 4 donors, Dunn’s). *P < 0.05, **P < 0.01. ^#^*P* < 0.05.

Human primary CD8^+^ T cells were precultured in control or high-NaCl or -urea conditions, which were also applied during the killing assay (experimental setup in Figure 6A). CFSE-stained human tubular epithelial cells were enumerated after coincubation. NaCl abolished tubular epithelial cell killing, while acute exposure did not significantly impair their cytotoxicity (Figure 6B). Very similar results were seen for urea (Figure 6C). Cytokines were measured in the supernatants (Figure 6, D and E). TNF-α, but not other T cell cytokines, was significantly suppressed by elevated NaCl concentration during initial CD8^+^ T cell culture but unaffected during restimulation (Figure 6, D and E).

To study specific antiviral response, CD137 upregulation in response to BKV peptide was assessed (45). Primary human T cells were investigated after culture with BKV peptide in total PBMCs required for presence of antigen-presenting cells (Figure 6F). The proportion of CD137^hi^ responder CD8^+^ T cell increased after stimulation, with the expected wide range of absolute values in different donors (paired Student’s 2-tailed *t* test). CD137^hi^ response was significantly blunted after culture with additional NaCl or urea (Figure 6G). This is consistent with its trend toward mRNA downregulation after allogeneic stimulation (Supplemental Figure 14A). Combinations of NaCl and urea, tested in the same total osmolarity and the same individual concentrations, suppressed CD137 even more, suggesting cooperative effects (Figure 6G). NaCl similarly impaired response to EBV peptide (Supplemental Figure 15). Culture supernatants were assessed for BKV peptide–induced cytokine production. Typical secondary mediators of dendritic cells were detectable. Among mediators significantly regulated by BKV peptide in control conditions (Figure 6, H–J), differential patterns were observed. IFN-γ was similarly upregulated by BKV peptide in elevated NaCl and in control but not in urea (Figure 6H). IFN-γ, IL-1β, IL-8, IL-18, and CCL2 baseline levels in NaCl tended to be higher than in controls and to decrease in urea (Figure 6, H–J). This pattern was previously reported for CCL2 in human monocyte-derived macrophages (24). While IL-1β, IL-8, IL-18, and CCL2 increased in response to BKV peptide in control conditions, there was a blunted response with added NaCl. With added urea, principal cytokine regulation by BKV peptide appeared to be sustained (Figure 6, H and J). Consistent trends were observed with EBV peptide (Supplemental Figure 15B) and for *IL1b* and *CCL2* gene expression in allogeneically stimulated CD8^+^ T cells (Supplemental Figure 14, B and C). It is of note that other mediators, including TNF-α, were below the detection limit, likely because of the low number of BKV peptide–reactive T cells.

To start to investigate the situation in the human kidney, primary CD8^+^ T cells from histologically healthy human kidney cortex and medulla were studied after culture in elevated NaCl and in control conditions. Also here, NaCl significantly decreased cortical but not medullary BKV-reactive CD8^+^ T cell numbers (Figure 6K).

These data showing impaired allogeneic cell killing, response to BKV peptide, and secondary cytokine production provide a mechanistic basis for how the osmolyte gradient impacts local T cell response.

## Discussion

Our data demonstrate impaired CD8^+^ T cell numbers and function in the presence of elevated NaCl and urea renal osmolytes, providing a mechanistic base to decreased BK viremia and improved viruria outcome with gradient-depleting diuretic therapy in the Ktx recipient cohort reported here. Together with differential CD8^+^ T cell densities in human and murine healthy kidney compartments, our data establish NaCl and urea as regulators of renal CD8^+^ T cell responses.

In our patient cohort, loop diuretic treatment was underrepresented in the BK viremic group. BK viremia is broadly considered an indicator of nephropathy (5, 8). While loop diuretic therapy did not associate with isolated viruria, a better kidney functional development was observed in viruric patients who received a loop diuretic. It is conceivable that viral spread from the urinary compartment to the circulation, i.e., viremia development, may indicate a high amount of virus liberated from tubular cells secondary to an insufficient immune response and associated damage. Among known risk factors for BKV reactivation during the first year after Ktx, our cohort with a relatively uniform immunosuppressive regimen of CNI, mycophenolate as antiproliferative agent, and long-term low-dose steroids, who underwent routine screening in blood and urine, experienced viremia and biopsy-confirmed BKVn rates somewhat lower than recent similar cohorts (5, 46) and higher than a meta-analysis of controlled interventions (6). Outcomes also confirm the course of BKVn after early detection. Living or deceased donor, the use of tacrolimus or cyclosporine as CNI, or rejections, which influenced risk to varying degrees in other studies (6), did not significantly contribute to BKV risk in our cohort. Our data confirm male sex as a risk factor of BK viremia (5, 6, 47). Similar protective trends of loop diuretic use were found in men and women. While containing clinical heterogeneity and waiting to be independently confirmed, the protective effect of loop diuretic use was maintained in multivariable analysis and propensity score matching including known risk factors.

Our data demonstrate lower CD8^+^ T cell densities in human and murine kidney medulla than cortex and verify the presence of BKV-reactive T cells in normal human kidney (48). Elevated NaCl induced CD8^+^ T cell death characterized by phosphatidylserine externalization and caspase-3 activation as hallmarks of apoptosis (49). This was not elicited by mannitol tonicity control and is reminiscent of myeloid cell death induced by even higher concentrations of NaCl (27). To start to define underlying pathways, our study determined that blockade of upregulated Na^+^-transporter SNAT2 (*SLC38A*) prevented early caspase-3 activation and that inhibition of p53, an upregulator of osmolyte-induced *AEN*, at least temporarily prevented CD8^+^ T cell death. Our findings on CD8^+^ T cell death in elevated NaCl agree with an FDGB-PET study on activated lymphocyte homing in a rat model of renal allograft rejection where fewer live cells were detected in medulla than cortex (50). During BKV reactivation after Ktx, a de novo T cell response by the recipient by cells from normotonic environments will be required, as the virus is nearly exclusively of donor origin (51). Our data show that endogenous medullary BKV-reactive CD8^+^ T cells tolerated an elevated NaCl concentration upon restimulation better than their cortical counterparts. This may indicate previous adaptation processes, which remain to be defined.

Our results indicate that elevated NaCl and urea concentrations impair CD8^+^ T cell activation by viral peptides from BKV and in some aspects also EBV, pointing at a more general effect on antiviral response, allogeneic tubular epithelial cell killing, and TNF-α production, in addition to diminishing their numbers. Indeed, BKV reactivation was a major side effect of TNF-α inhibition by infliximab after Ktx (36). Among secondary mediators of T cell activation depressed in a NaCl-rich environment, CCL2, which is markedly upregulated in urine during BKVn (52), was most prominent, despite its constitutive upregulation by NaCl in myeloid cells (24). Indeed, the overall effect of both NaCl and urea on antiviral response in the kidney tested here differs from antibacterial effects studied earlier (23, 24, 27). This is consistent with the fact that antiviral response requires memory formation (10), while bacterial infections are mainly controlled by short-lived innate immune cells (53).

Osmolytes may also impact other renal adaptive immune processes, some of which are also accessible to deeper mechanistic studies in experimental animals. In humans, this includes localization of kidney graft rejection, which mostly is considered a cortical rather than medullary condition. As IFN-related genes were downregulated by NaCl and by urea, the relevance of NaCl and urea should be investigated in other medullary infections, such as Hantavirus nephropathy, as well as tubulointerstitial healing processes (54). In vivo quantification of the medullary concentration gradient, which can be performed by MRI for sodium, together with spatial transcriptomics and proteomics of biopsy tissues, should be considered. Loop diuretic effects on volume and electrolyte status need to be monitored closely. Gradient assessment and possibly modulation may also be of interest during experimental therapies with anti-BKV T cells (55).

In summary, our data propose an improved antiviral T cell response at lower NaCl concentrations, including loop diuretic therapy during early-stage BKV reactivation. This is amenable to testable therapeutic interventions.

## Methods

### Sex as a biological variable.

The transplant cohort consists of males and females, and potential differences were explored but not apparent. The murine experiments were therefore conducted in only females.

### Human native kidney tissues and Ktx recipients.

Adult renal transplant recipients at Hannover Medical School (MHH) during 10 years starting with commencement of systematic BKV screening in serum and urine (2008–2017) who attended the outpatient clinic at least once (*n* = 1,461) were included if they received follow-up at this institution for at least 12 months and at least 2 complete BKV screenings (*n* = 772), one of them between month 12 and 15 (*n* = 649). Clinical chemistry values were assessed in the MHH clinical laboratories. BK viremia and viruria were analyzed by PCR, and 2,000 IU/mL and above was considered positive. Immunosuppressant and diuretic use and dose (furosemide or torasemide; torasemide dose was multiplied by 4 to express the biological equivalent) at first outpatient presentation were extracted from the clinical records. 96.5% of the loop diuretic nonrecipients remained without loop diuretic, and 97% of the loop diuretic group remained on loop diuretic for the observation period, resulting in mean daily doses of 60 ± 56 mg at start and 52 ± 50 mg at the end. For all participants transplanted in 2012 and 2013 (*n* = 130), a time period when 3-month surveillance biopsies were performed, biopsies obtained 45–180 days after Ktx (*n* = 86) were searched in the archives for reanalysis according to the current Banff criteria and additional immunohistology (*n* = 65 with sufficient material). Healthy tissues from partial tumor nephrectomies from patients without diuretic prescription were identified in the archives and included in the analysis if they contained both cortex and medulla (*n* = 6, 67% females, age 72 ± 8.5 years). Immunostaining and microscopy of kidney tissues and quantification of renal immune cell infiltrates are detailed in the online Supplemental Methods.

### Mice.

Female C57BL/6 mice (The Jackson Laboratory, bred locally) were housed in an environment with controlled temperature and humidity, a 12-hour light/12-hour dark cycle, and access to water and food ad libitum. Mice were given furosemide intraperitoneally twice daily (15 mg/kg) starting 4 days before sacrifice.

### Primary human and murine blood and kidney cell isolation, cell culture, and flow cytometry.

Isolation and stimulation protocols as well as analysis methods are detailed in the online Supplemental Methods.

### RNA sequencing.

RNA isolation, sequencing at NGS Core Facility Bonn, and subsequent analysis are detailed in Supplemental Methods. Gene counts were normalized to total reads per sample, which did not differ for the conditions or donors (Supplemental Figure 9B). Genes were included in the analysis if expressed in at least 1 control sample. Regulated gene groups were visualized using Swissprot String database at https://string-db.org, showing all types of interactions of connected genes only (56). Markov clustering was performed with an inflation parameter of 1.5. Gene expression in murine kidney cortex, and outer and inner stripe of outer medulla, the region of highest expression, was extracted from GSE81741, published before (57) (*n* = 3 healthy mice). Gene expression data from human naive CD8^+^ T cells stimulated with anti-CD3 and anti-CD28 with (35) or without (34) exogenous IL-2 in the absence and presence of additional 80 mmol (35) or 67 mmol (34) (GSE232365) were analyzed. For all datasets, with the aim of hypothesis generation, genes up- or downregulated at least 2-fold by NaCl with a raw *P* < 0.05 were included in the analyses.

### Statistics.

Mechanistic in vitro experiments in exploratory setups without prior knowledge of result variability in primary human cells were typically performed with *n* = 4–8 depending on variance of findings. In principle, normality was assumed for continuous biological variables because most follow a Gaussian distribution. Normal distribution was tested by d’Agostino-Pearson test if numbers allowed. Two-tailed Student’s *t* test with Welch’s correction if variance was unequal or Mann-Whitney for nonparametric values was used to compare 2 conditions, and correlation and logistic regression analyses were calculated using GraphPad Prism. If more than 2 conditions were compared, Dunnett’s test was applied after 1-way ANOVA or nonparametric test was employed as appropriate and indicated in the figure legends.

Propensity score matching was performed using R (version 4.3.1 in RStudio 2025.05.1+513) with the *MatchIt* and *cobalt* packages using a logistic regression model including age at transplantation, sex, cold ischemia time, donor type (living vs. deceased), HLA mismatches, ABO incompatibility, CNI use, tacrolimus use, mTOR inhibitor use, baseline eGFR, DGF, and occurrence of rejection. Nearest neighbor matching (1:1) with a caliper of 0.1 of the SD of the logit of the propensity score was conducted, resulting in 143 matched pairs. Standardized mean difference (SMDs) for all the covariates, except for tacrolimus, were below the 0.1 threshold. Tacrolimus use (SMD = 0.176) was included as a covariate in the outcome model.

Data are expressed as mean ± SEM unless indicated otherwise. *P* values < 0.05 were considered significant and are indicated.

### Study approval.

Human renal and blood leukocytes were analyzed after receipt of written informed consent and local ethics board approval (MHH 807, UKB 039/22). Kidney transplantations were conducted according to the Declaration of Istanbul. The ethics board approved of and waived informed consent for the retrospective study on archived samples (MHH 3516, 10183). Animal experiments were approved by Landesamt für Natur-, Umwelt- und Verbraucherschutz, Recklinghausen, Nordrhein-Westphalia, Germany, in accordance with the guidelines from Directive 2010/63/EU of the European Parliament on the protection of animals used for scientific purposes (#81-02.04.2018.A089).

### Data availability.

Gene expression datasets have been uploaded to NCBI GEO (GSE279307). Values for all data points found in graphs are in the Supporting Data Values file. Please find Supplemental Methods in the online supplement.

## Author contributions

PF, AG, LO, JS, CK, JM, HH, and SVV designed research. AG, JS, JM, and OB performed experiments. PF, AG, and LO acquired data. NK recruited patients. PF, JS, JHB, and MT evaluated renal histology. PF, AG, IG, and SVV analyzed data. PF, AG and SVV wrote the manuscript with help from LO, JS, JM, IG, JHB, NK, OB, CK, HH, and MT. All authors read and approved the manuscript. First author order is alphabetical.

## Supplementary Material

Supplemental data

Supporting data values

## Figures and Tables

**Table 1:**
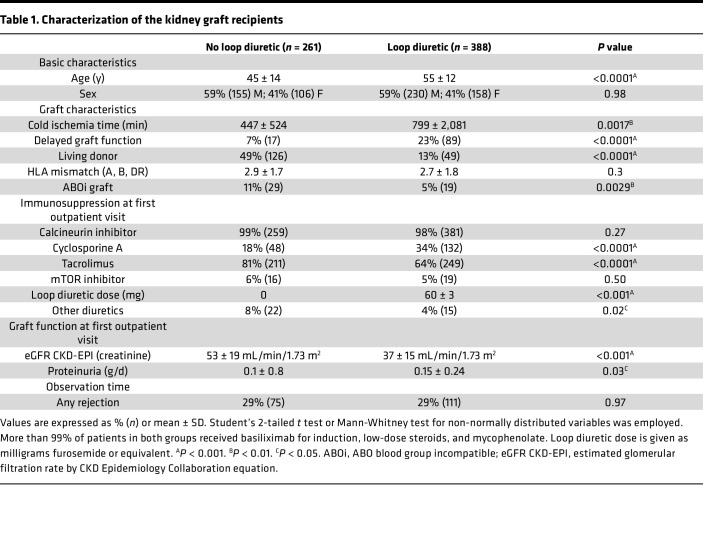
Characterization of the kidney graft recipients

**Table 2:**
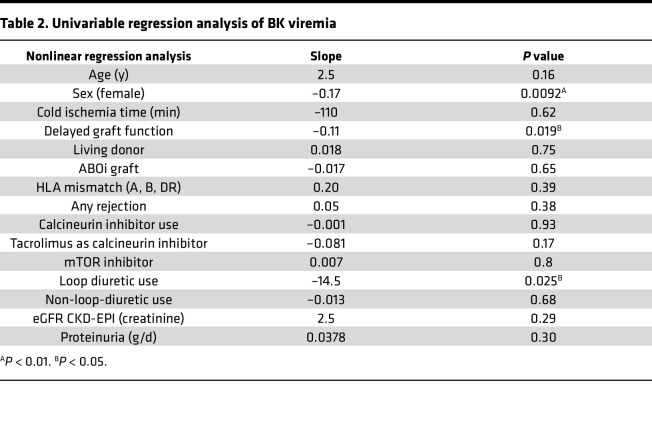
Univariable regression analysis of BK viremia

## Version 1 08/26/2025

In-Press Preview

## Version 2 10/08/2025

Electronic publication
