# Supplementary material for: NaCl and urea modulate CD8+ T cell survival, renal accumulation, and response to BK virus
Source: JCI Insight. 2025 Aug 26;10(19):e194570. doi: 10.1172/jci.insight.194570 (PMC12513482; doi:10.1172/jci.insight.194570)
Supplement: Supplemental data [file jciinsight-10-194570-s089.pdf]

Supplemental Information on:

Peyman Falahat, Adrian Goldspink, Lucia Oehler, Jessica Schmitz, Julia Miranda, Islem Gammoudi, Jan Hinrich Bräsen, Niklas Klümper, Olena Babyak, Christian Kurts, Herrmann Haller, Marieta Toma, and Sibylle von Vietinghoff

NaCl and urea modulate CD8$^+$ T cell survival, renal accumulation and response to BK virus

Contents:

Supplemental methods

Supplemental Figures 1-16

Supplemental Tables 1-8

Supplemental Methods

Immunostaining and microscopy of kidney tissue

Paraffin sections of murine kidneys was stained with rabbit anti-mouse CD8a (1:1000, Abcam) after heat-pretreatment (pH 9.0, Tris/EDTA buffer), quenching with Peroxidase Suppressor (Thermo Fisher) and blocking with universal blocking buffer (Thermo Fisher) over night at 4 °C followed by HRP-conjugated anti-rabbit IgG (ab6721, Abcam) DAB/Metal staining (Thermo Fisher) using Pierce™ Peroxidase Detection Kit (Thermo Fisher) with hematoxylin counterstain.

Human kidney transplant biopsies were fully re-scored according the Banff 2022 classification by an experienced nephropathologist (JHB)(1). Immunostaining of serial sections with polyclonal rabbit anti-CD3 (Agilent, 1:100), monoclonal rabbit anti-CD4 (SP35, Zytomed Systems, 1:50), monoclonal mouse anti-CD8 (C8/144B, Agilent, 1:50) and monoclonal rat anti-CD209 (h209, LSBio, 1:50) antibodies on heat-pretreated (Zytomed Systems) rehydrated sections (CD3, CD4, CD8: TRIS-EDTA buffer, pH 9; CD209: citrate buffer, pH 6, 98°C, 40 min) using donkey anti-rabbit IgG and donkey anti-mouse IgG (both from Jackson ImmunoResearch) or anti-rat IgG VisUCyte HRP Polymer antibodies (R&D Systems), respectively, after peroxidase blocking (3% H_2O_2) was essentially as described (2). Chromogen detection was performed with 3,3'-diaminobenzidine (Zytomed Systems) and hemalum counterstain. Negative controls omitting primary antibody were included into all staining protocols.

Quantification of renal immune cell infiltrates

Whole slide images were digitized with Aperio CS2 (Leica Biosystems) at 20x magnification for murine and Metafer Scanning Platform (MetaSystems) at 20x magnification for human samples. QuPath (v0.4.4 and 0.5.1) was used for quantitative scan analysis (3, 4). The reviewer of all murine and human histologic stainings was blinded to diuretic therapy and any clinical

data and only given an identification number, scan order was randomized. Human medulla and cortex were identified by the presence and absence of glomeruli, facilitated by frequently visible vasculature between these regions. Identification of murine cortex was similar, inner and outer medulla were distinguished by tubular epithelial structure. Prior to applying a semi-automated positive cell detection approach, each scan was annotated for cortex or medulla using QuPath's wand tool, followed by the "fill-holes" function and a manual artefact check. For positive cell detection, a pixel size of 0.1µm, a background radius of 6µm, a sigma of 2µm, a minimum area of 10µm^2, and a maximum area of 400µm^2 were requested. At least 5 positively stained cells were averaged for threshold definition. Scans were analyzed for proportion of stained cells defined by nuclei.

Primary human blood and kidney cell isolation, murine kidney cell isolation, cell culture and hyperosmolar stimulation

Human peripheral blood mononuclear cells from anonymized buffy coats (PBMC) were enriched by density gradient centrifugation with Pancoll 1.077 (PAN-Biotech) as described (5). Normal kidney cortex and medulla tissues were dissected by a certified pathologist (MT) from kidneys of male patients, age 67 (56-73) years, who underwent partial nephrectomy for renal cell cancer at UKB urology department, and cultured immediately after enzymatic digestion as described (6). None of the patients received diuretic therapy at the time of surgery. Non-adherent cells collected after 2h culture in full Roswell Park Memorial Institute (RPMI, 296mOsm/L, 138.4 mM Na^+) 1640 media with GlutaMAX™ (Thermo Fisher Scientific) supplemented with 10%FCS (PAN-Biotech). Outer and inner medulla were dissected and filtered through a 70µm cell strainer (Corning). HK2 human tubular epithelial cells (Cell Lines Service) were cultured in full Dulbecco's Modified Eagle Medium: F12 (DMEM, 328mOsm/L, 150.5 mM Na^+, Lonza) supplemented with 10% FCS and were let to adhere to tissue culture plate for at least 2h prior to addition of PBMC. Co-culture of HK2 cells with blood leukocytes

was in 50% RPMI/50% DMEM (144.4 mM Na^+). NaCl, urea, Pifithrin (30 µM, Sigma Aldrich, solvent control: DMSO 1:3333) or $HgCl_2$ (10µM, Sigma Aldrich, dissolved in PBS), were added in the indicated concentrations. Cells were transferred onto fresh HK2 cells for restimulation. Carboxyfluorescein succinimidyl ester (CFSE, 5µM, Cayman Chemicals), PepTivator® BKV or EBV (10µl/5+10^6 cells) and $CD8^+$ T Cell Isolation Kit (purity 81±3.7% $CD8^+$/$CD3^+$ cells in 8 indep. isolations, both: Miltenyi) were employed according to the manufacturers' instructions.

Flow cytometry

The following antibodies were used: anti-mouse: anti-CD3 (17A2), anti-CD4 (GK1.5), anti-CD8 (53-6.7) anti-CD11b (M1/70), anti-CD19 (6D5), anti-CD45 (30-F11) (Biolegend), anti-human: anti-CD3 (HIT3a), anti-CD8 (SK1, HIT8a), anti-CD11b (ICRF44), anti-CD27 (M-T271), anti-CD54 (HA58), anti-CD137 (4B4-1), anti-ITGb7 (FIB504), anti-KLRG1 (SA231A2) (Biolegend). AnnexinV (Biolegend), near-infrared LIVE/DEAD® Fixable Dead Cell Stain Kit (both: Invitrogen) and FITC Active Caspase3 Apoptosis Kit (BD Bioscience) were used according to the manufacturers' instructions. Human counts were determined by BD Calibrite™ APC Beads (BD Bioscience) at a final concentration of 10000 beads/ml, murine kidney cells counts using BD Truecount™ Tubes (BD Bioscience). Cytokine concentrations were analyzed with LEGENDplex™ Human inflammation Panel 1 (Biolegend) in supernatants stored at -80°C until analysis. Flow cytometry was performed on a Becton-Dickinson FACSCanto or LSR Fortessa Cell Analyzer (BD Bioscience), cell sorting on a BD FACSAria III Cell Sorter (BD Bioscience) or Sony MA900 Multi-Application Cell Sorter (Sony Biotechnology, San Jose, CA) at 4°C. Data were analyzed using FlowJo software (Tree Star Inc.).

RNA sequencing

RNA was isolated using NucleoSpin RNA Kit (Macherey-Nagel), yield and purity were determined in a NanoDrop 1000 Spectrophotometer (Thermo Scientific). RNA samples were sequenced at NGS Core Facility Bonn. The 3'mRNA Seq was performed using the Lexogen QuantSeq 3'mRNA FWD Library Prep Kit using 1000ng total RNA as input material. Sequencing was performed on an Illumina NovaSeq 6000 device at 10M reads per sample. Data was processed using nf-core/rnaseq v3.14.0 (doi: https://doi.org/10.5281/zenodo.1400710) of the nf-core collection of workflows (7), utilising reproducible software environments from the Bioconda (8) (and Biocontainers (9) projects. The pipeline was executed with Nextflow v24.04.2(10) with the following command: nextflow run nf-core/rnaseq -profile docker -params-file params_human.yaml.

Supplemental Figures:

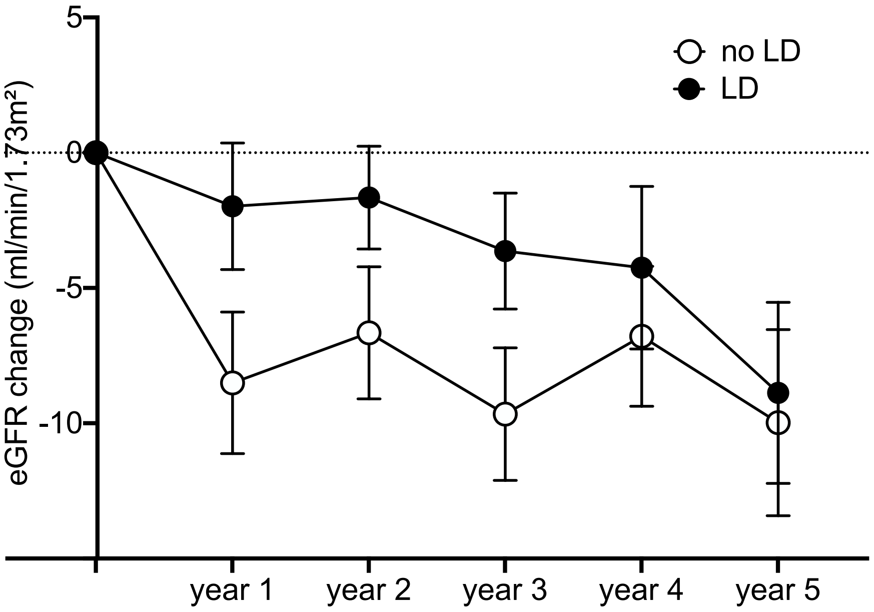

**Number at risk**					
No LD	28	28	27	23	18
LD	28	26	22	17	16

Supplemental Figure 1: eGFR development after diagnosis of BK viremia

The change in eGFR calculated by the creatinine-based CKD-EPI formula in patients clinically diagnosed as BKV (i.e. viremia) during the first twelve month over five years after kidney transplantation according to loop diuretic (LD) use (indicated with black versus white circles, stacked mixed-effects ANOVA: significant for time ($p<0.0001$), non-significant for LD use ($p= 0.154$)). Number of patients at risk is indicated at the bottom of the figure, whiskers indicate SEM.

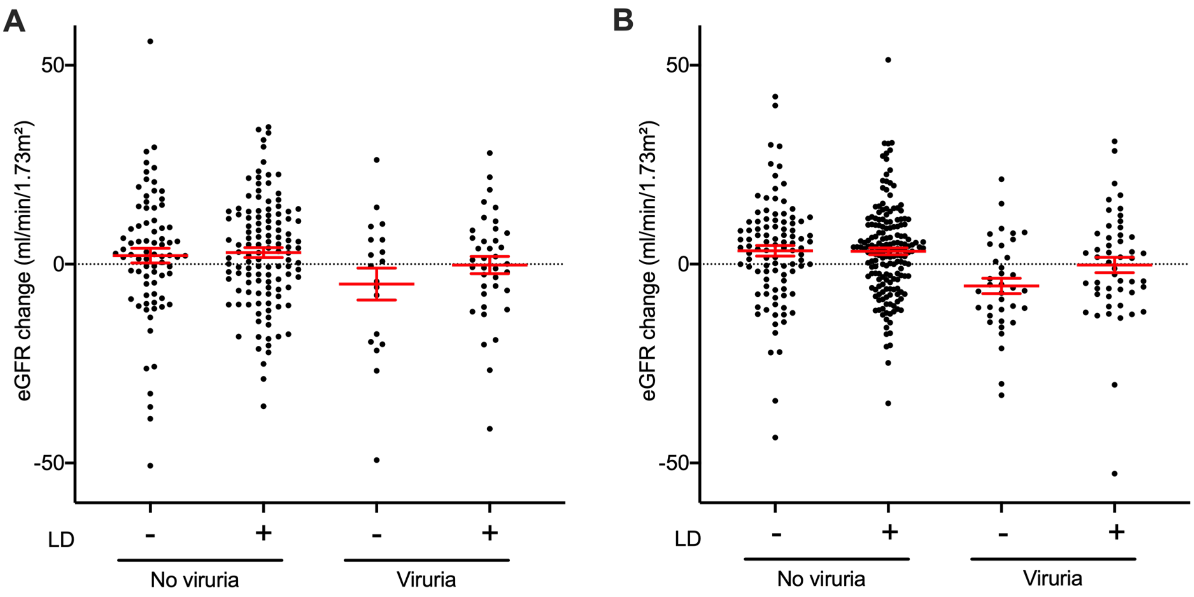

Supplemental Figure 2: eGFR development with and without viruria and loop diuretic therapy in females and males

(A,B) eGFR development during the first year after kidney transplantation in the subgroups of female (A) and male (B) patients with and without BK viruria and loop diuretic (LD) therapy.

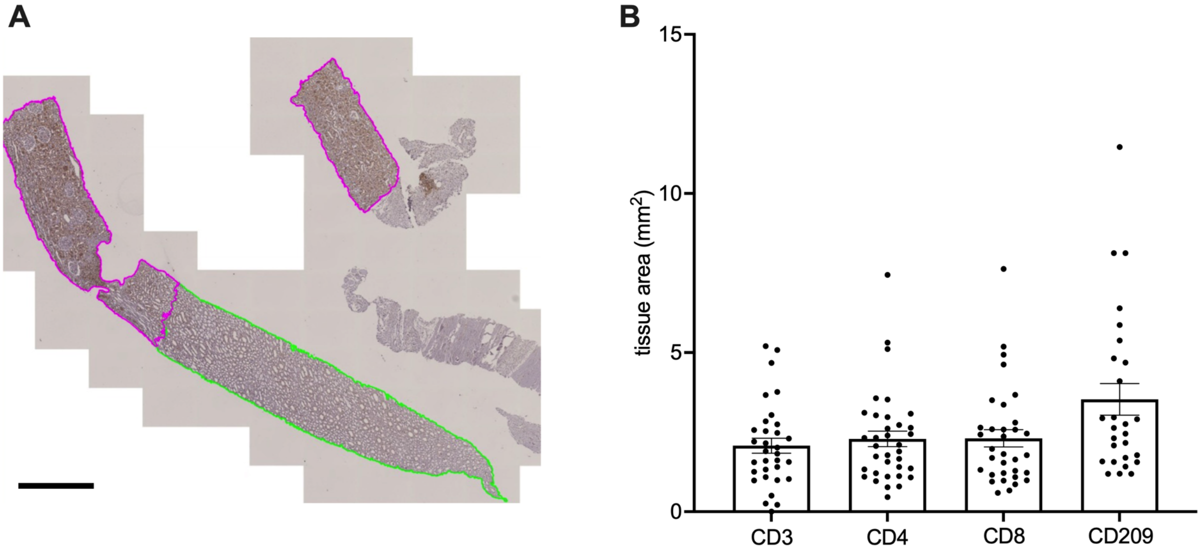

Supplemental Figure 3: Quantification of medullary areas in whole slide scans of kidney biopsies

(A-C) Renal cortex (pink) and medulla (green) were annotated on whole-slide scans of the kidney-biopsy specimen (A, size bar indicates 1mm). **(B)** Size of medullary tissue areas that were evaluated for each patient and staining (Dunn's).

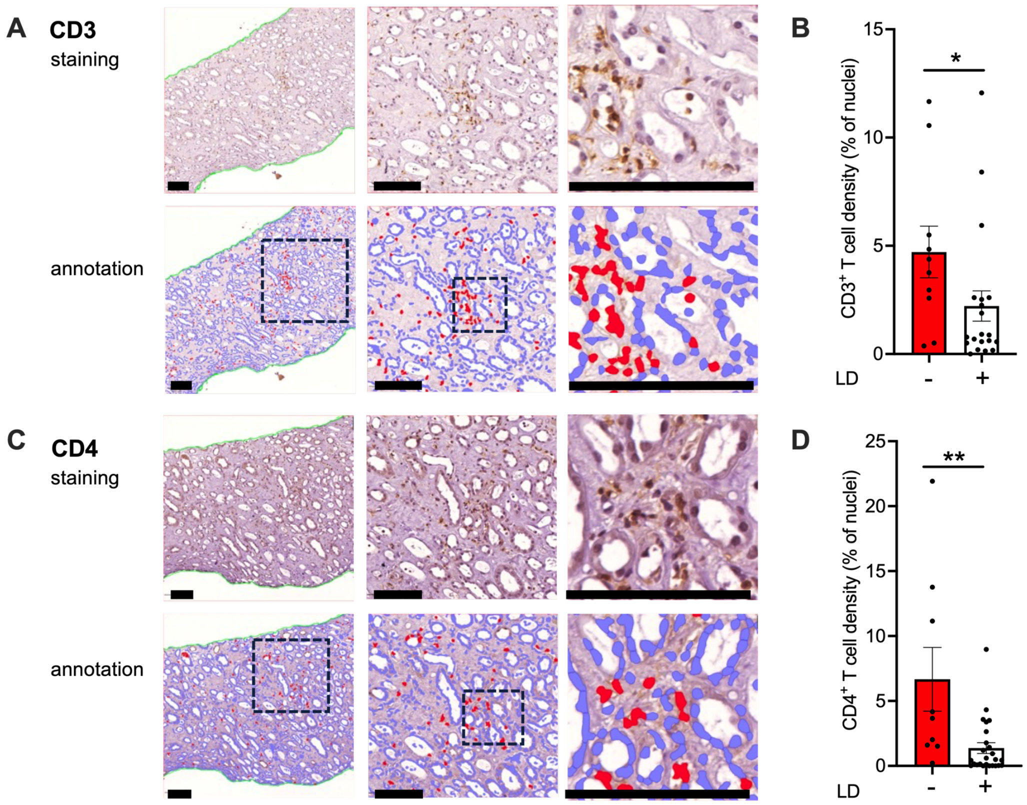

Supplemental Figure 4: Loop diuretic therapy association with T cell densities in the renal allograft

(A-D) Total $CD3^+$ (A,B) and $CD4^+$ helper (C,D) densities in three-month post-implantation biopsies of kidney allografts according to loop diuretic (LD) use (A,C examples of stainings and annotations of typical sections, bars indicate 100µm, B,D statistical analysis by Mann-Whitney tests).

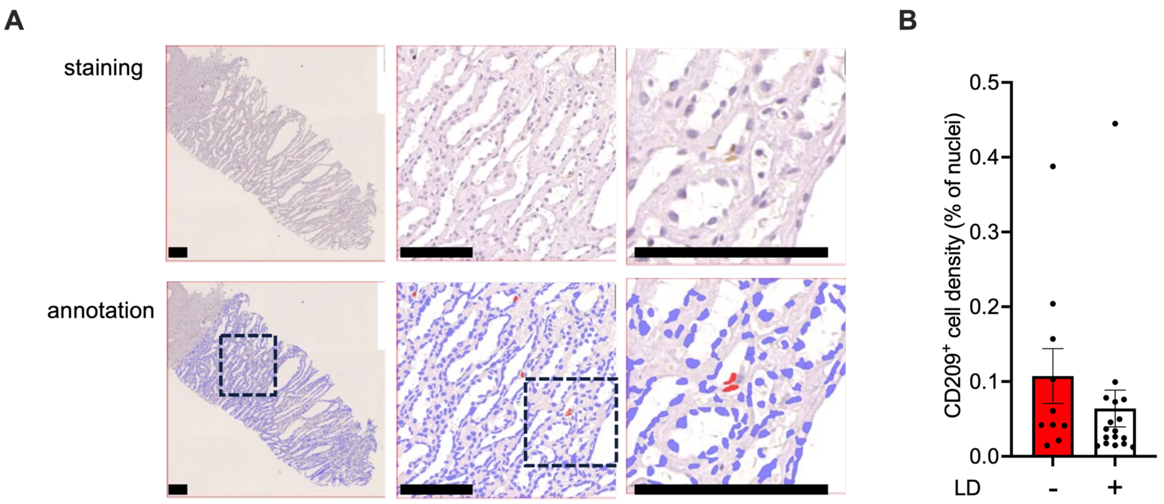

Supplemental Figure 5: Role of loop diuretic therapy for CD209 $^{+}$ ***antigen presenting cell densities in the renal allograft***

(A,B) DC-SIGN (CD209)$^{+}$ antigen presenting cell densities in three-month post-implantation biopsies of kidney allografts according to loop diuretic (LD) use (A, examples of stainings and annotations of typical sections, bars indicate 100µm, and B, statistical analysis by Mann-Whitney test).

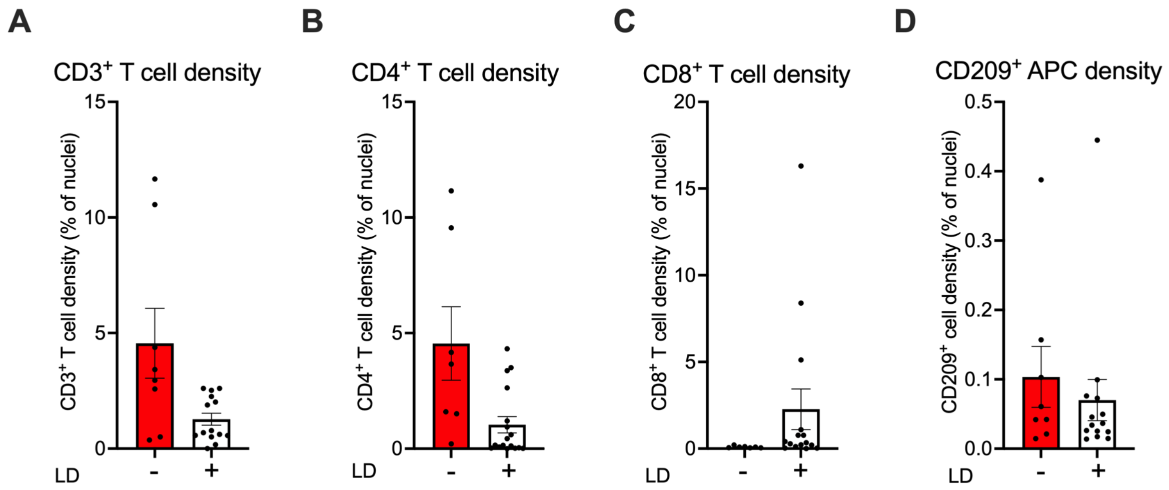

Supplemental Figure 6: Renal medullary leukocyte densities in the absence of rejection

(A-D) Densities of total $CD3^+$ (A) and $CD4^+$ helper (B), $CD8^+$ cytotoxic T cells (C) and $CD209^+$ antigen presenting cells (D) in biopsies without rejection or borderline rejection diagnosis according to loop diuretic (LD) use.

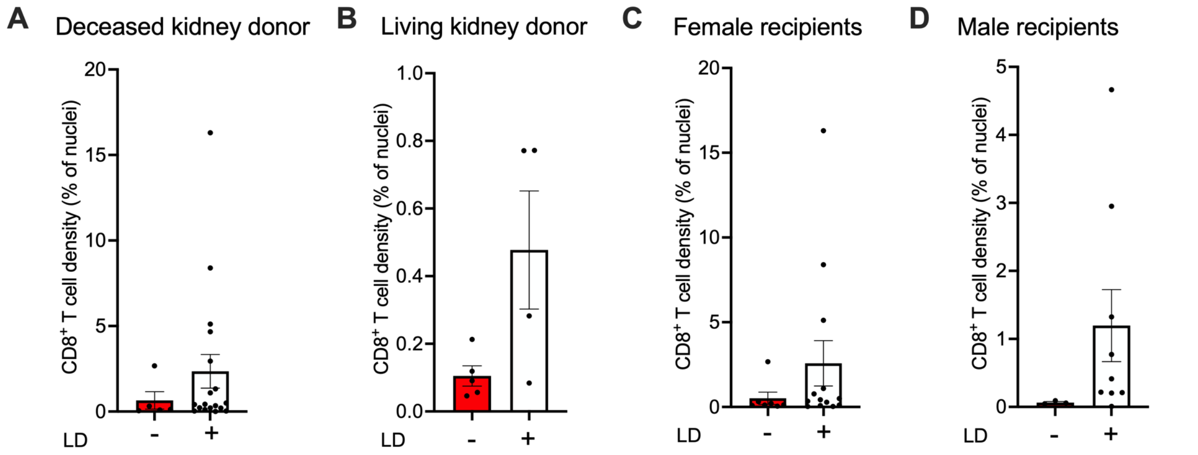

Supplemental Figure 7: Renal medullary CD8+ T cell densities depending on graft provenience and recipient gender

(A,B) Densities of $CD8^+$ cytotoxic T cells in biopsies from deceased (A) and living donor (B) kidneys and **(C,D)** Densities of $CD8^+$ cytotoxic T cells in biopsies from grafts transplanted into female (A) and male (B) recipients according to loop diuretic (LD) use separately.

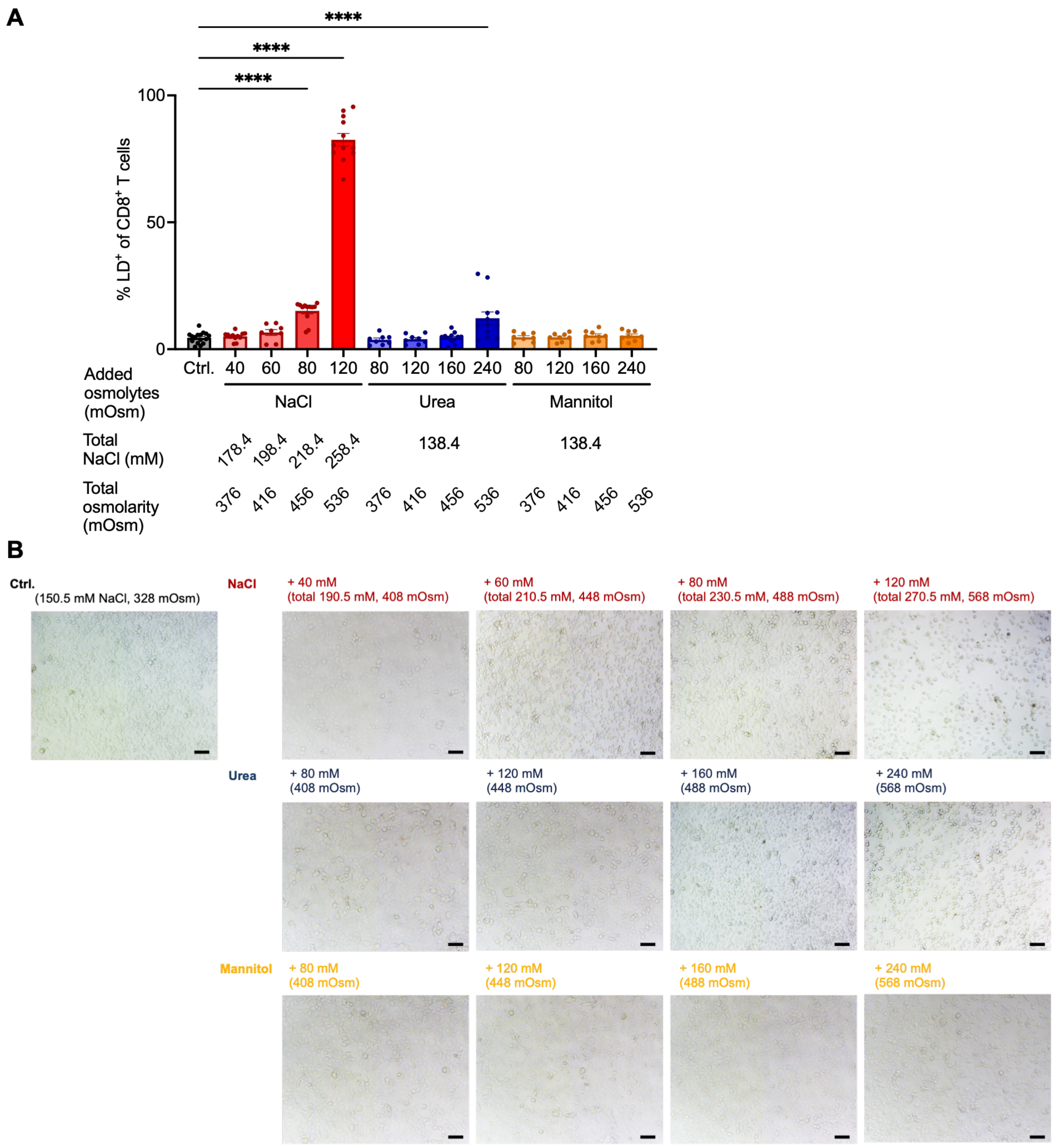

Supplemental Figure 8: T cell and tubular epithelial cell survival in elevated NaCl and urea

(A) Human adhesion depleted PBMC were cultured with allogenic stimulation by HK2 human tubular epithelial cells with and without addition of NaCl and urea or mannitol tonicity control in full RPMI media in the indicated concentrations resulting in the indicated total osmolarities. The proportion of dead (LD^+) $CD8^+$ T cells was enumerated by flow cytometry on day three (n=12 from 6 donors in 5 indep. experiments, Dunnett's after ANOVA). **(B)** HK2 cells were cultured for 20h with or without additional NaCl and urea or mannitol tonicity control in full DMEM media resulting in the indicated total osmolarities. Representative images of one of four experiments (bars indicate 100µm).

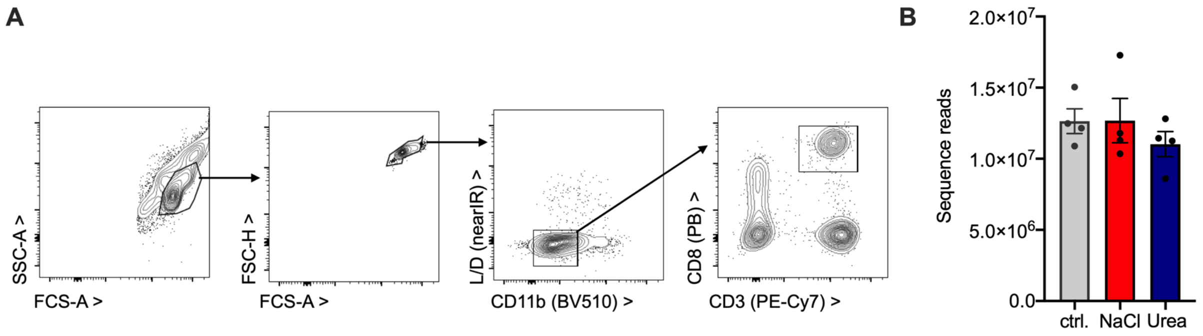

Supplemental Figure 9: Sorting for and read counts of CD8⁺ T cell RNA sequencing

(A) Gating strategy of flow cytometric live $CD8^+$ T cell sorting. $CD8^+CD3^+CD11b^-LD^-$ cells were selected for RNA isolation. **(B)** Sequence read counts did not differ between the three experimental groups (ANOVA).

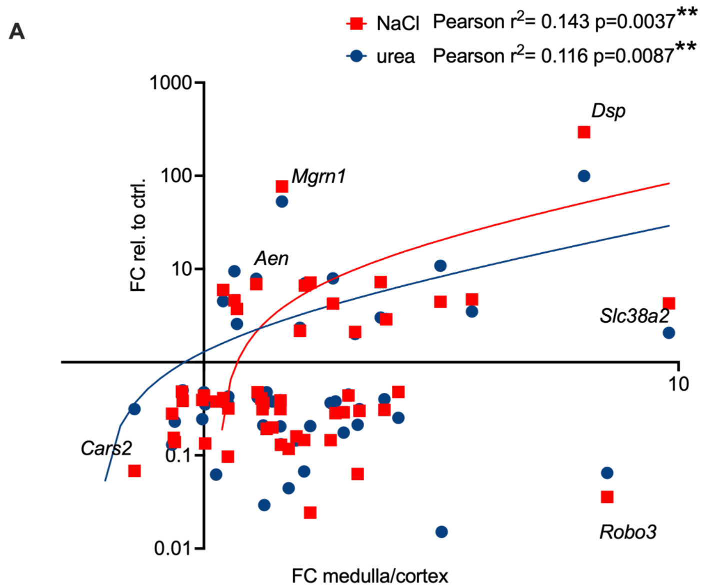

Supplemental Figure 10: Gene regulation correlation with the renal cortico-medullary gradient

Correlation of fold-change (FC) expression change of genes commonly regulated by NaCl and urea (n=72, defined in Figure 4F) with relative expression in healthy murine medulla versus cortex (n=3, GSE8174136, (11)).

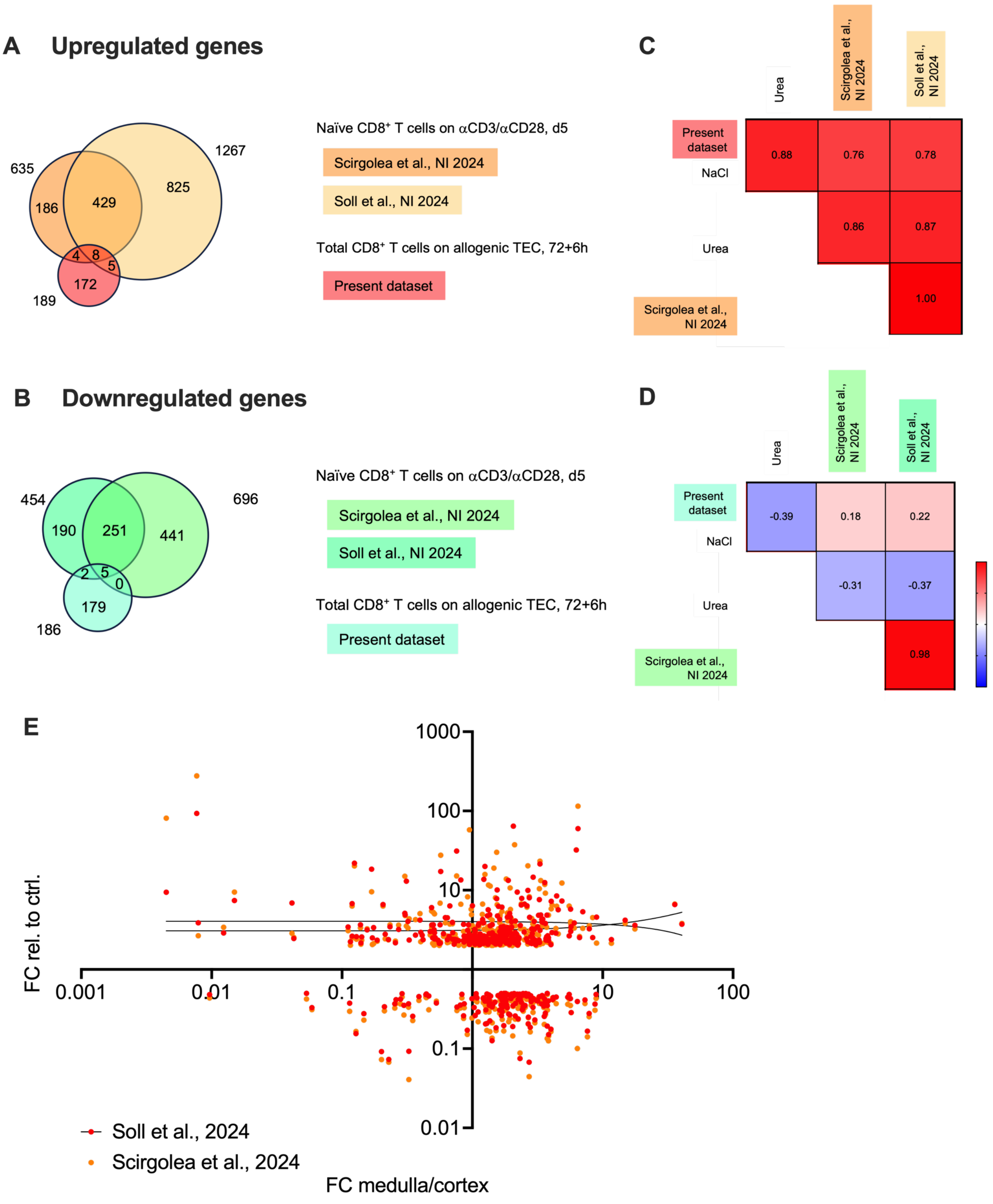

Supplemental Figure 11: CD8⁺ T cell gene regulation in comparison to the kidney cortico-medullary gene expression profile

(A-E) Gene expression profiles of published human naïve $CD8^+$ T cells stimulated with anti-CD3 and anti-CD28 with (12) or without (13) exogenous IL-2 in the absence and presence of additional 80 mmol (12) or 67 mmol (13) NaCl were compared with each other, our results in allogenic-restimulated total $CD8^+$ T cells and renal gene expression gradients. **(A,B)** Overlapping gene regulation among up- (A) and downregulated genes is visualized using Venn's diagram including genes with an at least twofold change. **(C,D)** Extent of up- (C) and downregulation (were) compared (Pearson's r, significance level obtained in all tests in C, and the downmost in D). **(E)** Correlation of gene regulation magnitude with the renal cortico-medullary gene expression gradient (11) for all commonly regulated genes in (12) and (13). No significant correlation was found (Pearson's test).

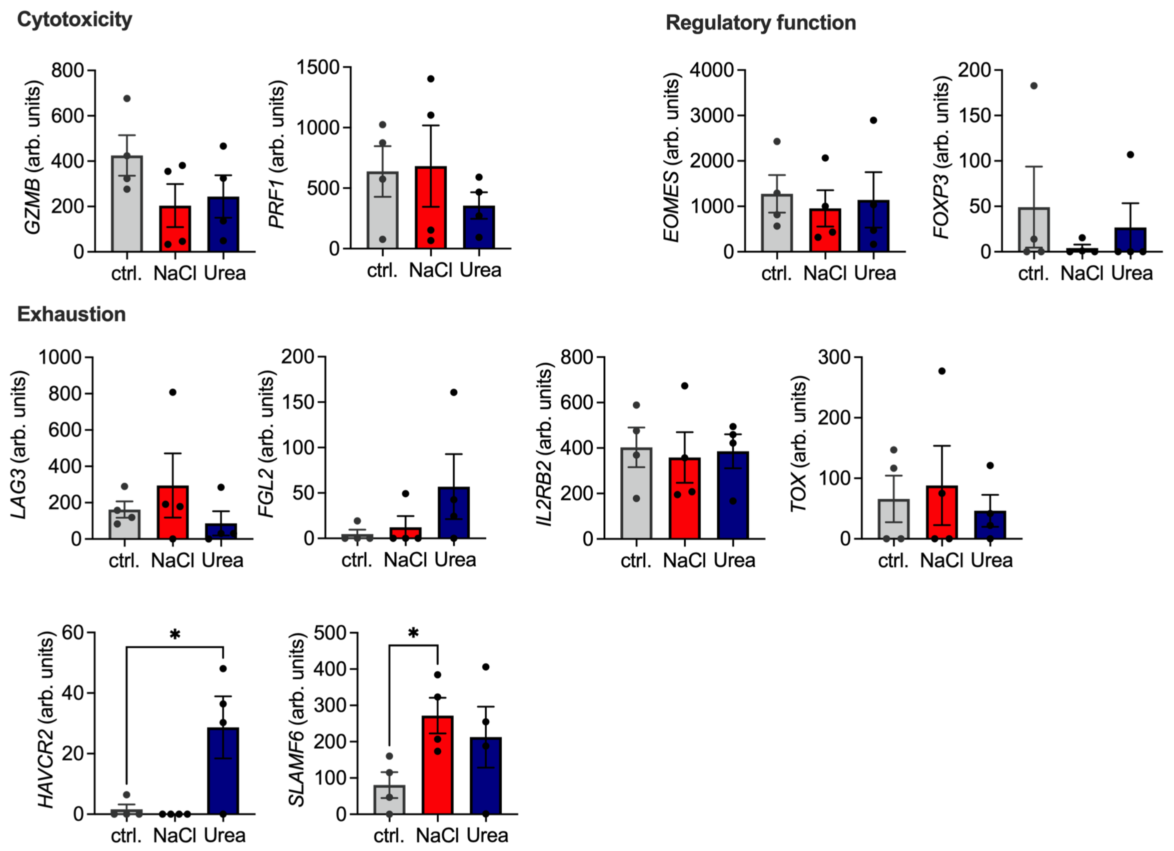

Supplemental Figure 12: Regulation of renal T cell activation marker expression by NaCl and urea

Markers used characterize T cell cytotoxicity, exhaustion and regulatory function in a recent study on chronic alloimmune response in the kidney,(14) and exhaustion markers *HAVCR2* (protein: Tim3) and *SLAM6F* were assessed in $CD8^{+}$ T cells isolated by flow cytometry by RNA sequencing as described in Figure 9 (n=4 donors, t-tests).

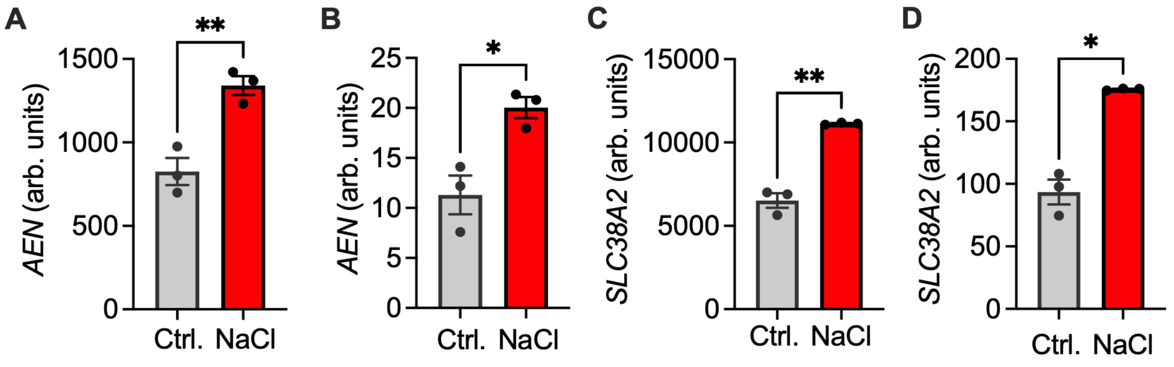

Supplemental Figure 13: AEN and SLC38A2 regulation in naive CD8⁺ T cells

(A-D) *AEN* (A,B) and *SLC38A*2 (C,D) gene expression profiles in human naïve $CD8^+$ T cells stimulated with anti-CD3 and anti-CD28 with(12) (A,C) or without(13) (B,D) exogenous IL-2 in the absence and presence of additional 80 mmol(12) or 67 mmol(13) NaCl (t-tests with Welch's correction).

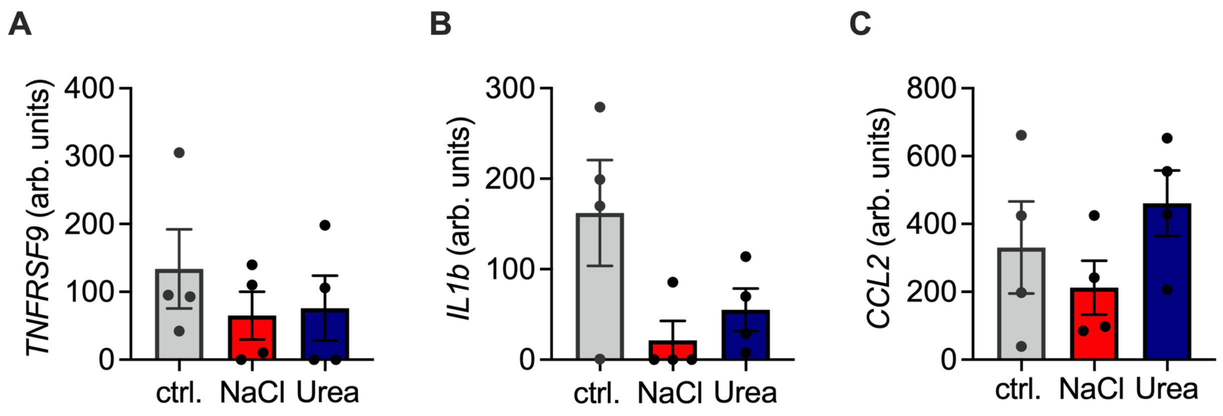

Supplemental Figure 14: Markers of T cell activation in CD8⁺ T cell RNA sequencing

(A-C) The CD137- coding gene *TNFRSF9* (A), *IL1B* (B) and *CCL2* (C) gene expression in isolated live $CD8^+$ T cells subjected to RNA sequencing (n=4 donors) as described in Figure 4. *IFNG, TNFA, IL8, IL10* and *IL18* were below detection limit.

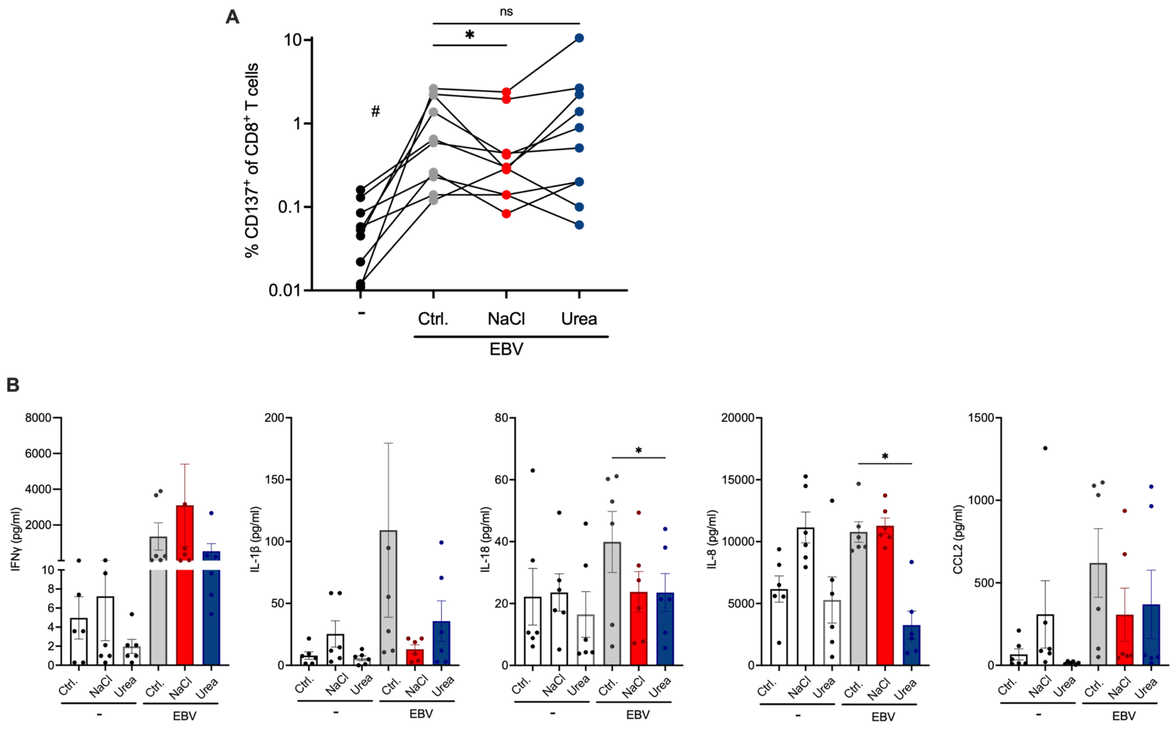

Supplemental Figure 15: Response to EBV-peptide is dampened by environmental NaCl

(A,B) PBMC were cultured with and without stimulation with EBV peptide in the absence or presence of additional 80 mM NaCl, equimolar 160 mM urea, or control for three days **(A)** The proportion of responsive CD137^{HIGH}CD8^{+} T cells was quantified by flow cytometry (gating in Supplemental Figure 9A, statistical analysis of n=10 from 5 donors in 3 indep. experiments, #paired t-test of stimulation, *Dunn's after ANOVA of peptide conditions). **(B)** Supernatant IFNγ, IL-1β, IL-18, IL-8, and CCL2 was assessed by cytometric bead assay (n=5 T cell donors in independent experiments, Dunn's of peptide conditions).

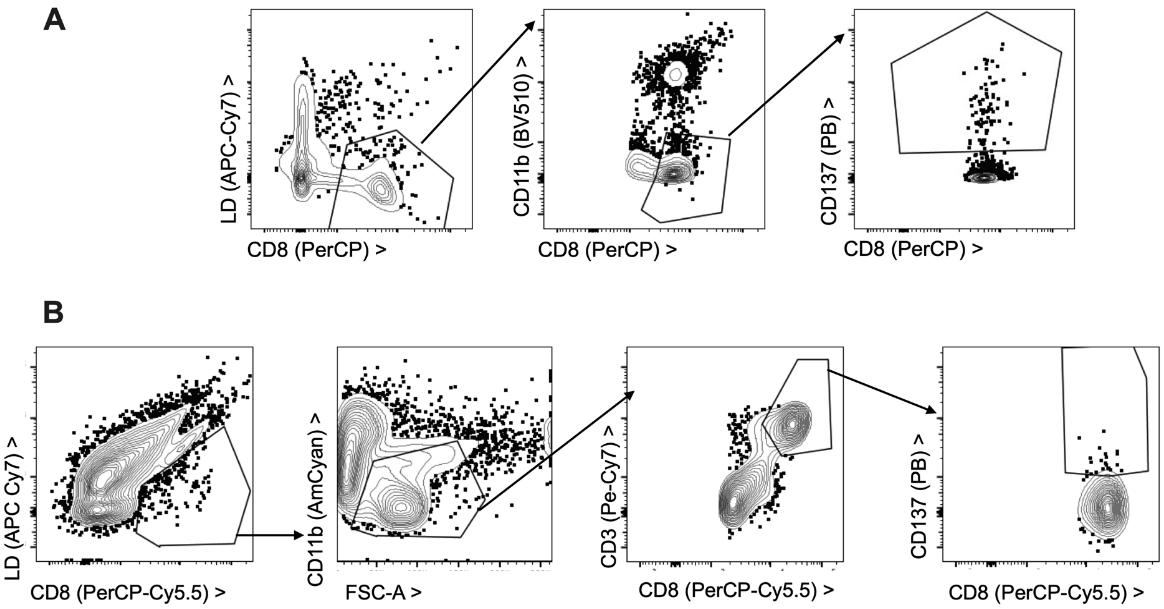

Supplemental Figure 16: Human CD8⁺ T cell gating strategies

(A,B) Live $CD8^{+}CD11b^{-}$ cells were gated from human PBMC (A) and live $CD8^{+}$ $CD3^{+}CD11b^{-}$ healthy human kidney tissues (B) to assess activation marker CD137 expression.

Supplemental Tables:

Supplemental Table 1: Therapy and outcome of BK viremic patients according to loop diuretic use

	**No loop diuretic (n=35)**	**Loop diuretic (n=31)**	**p-value**
**BKV**			
Maximal viral load (urine, IU/ml)	$2.2 \pm 4.2*10^7$	$8.3 \pm 17*10^7$	0.45
Maximal viral load (plasma, IU/ml)	$3.9 \pm 8.1*10^5$	$4.2 \pm 8.4*10^5$	0.96
Kidney biopsy with BKVn if performed	17% (2/12)	24% (6/25)	0.70
**Therapeutic measures**			
Mycophenolate dose reduction	97% (30/31)	86% (25/29)	0.19
Switch from mycophenolate to mTOR inhibitor	39% (12/31)	65% (17/26)	0.06
Calcineurin inhibitor cessation	30% (9/30)	40% (10/25)	0.57
High dose intravenous immunoglobulin	13% (4/31)	17% (5/29)	0.73
**Renal function**			
eGFR (ml/min/1.73m^2) at first outpatient visit	51.1±15	38.5±14	0.0029*

Therapeutic measures within two years after diagnosis are included.
Means±SD, Mann-Whitney tests for exploratory p-values.

Supplemental Table 2: eGFR development according to loop diuretic therapy, rejection status and BKV reactivation

**eGFR change**	**No LD**		**LD**		p-value
	ml/min/1.73m^2	n	ml/min/1.73m^2	n	
**All**					
All	0.88±15	231	2.3±13	386	0.33
No BKV	2.8±15	176	3.1±12	198	0.87
Viruria	-5.3±14	55	-0.2±13	88	0.02*
Viruria and viremia	-6.3±11	29	-3.2±15	30	0.17
**No rejection**					
All	1.1±15	163	2.7±13	276	0.29
No BKV	3.2±15	127	3.2±12	221	0.78
Viruria	-8.0±16	13	1.7±14	38	0.086
Viruria and viremia	-6.6±10	23	-1.7±17	17	0.15
**Any rejection**					
All	0.4±14	68	1.4±12	110	0.84
No BKV	1.8±13	49	2.8±12	77	0.85
Viruria	-0.3±16	13	0.6±8.5	10	0.96
Viruria and viremia	-9.2±14	6	-5.2±12	13	0.32

LD indicates loop diuretic therapy. Mean±SD, Mann-Whitney tests for exploratory p-values.

Supplemental Table 3: Characterization of the kidney graft recipients of the biopsy cohort

	**No loop diuretic (n=21)**	**Loop diuretic (n=44)**	**p-value**
**Basic characteristics**			
Age (years)	46±17	54±13	0.042*
Gender	57% (12) male	49% (22) male	0.6
**Graft characteristics**			
Cold ischemia time (min)	453±362	751±420	0.0078**
Delayed graft function	0% (0)	20% (9)	0.0475*
Living donor	52% (11)	9% (4)	0.0003***
HLA mismatch (A, B, DR)	3.1±2.3	2.6±1.6	0.28
ABOi graft	19% (4)	2% (1)	0.036*
**Immunosuppression at first outpatient visit**			
CNI	100% (21)	99% (42)	0.52
Cyclosporine A	19% (4)	33% (15)	0.26
Tacrolimus	81% (17)	67% (30)	0.26
mTOR inhibitor	0% (0)	0% (0)	1
Loop diuretic dose (mg)	0	69±46	<0.001***
**Graft function at first outpatient visit**			
eGFR CKD-EPI (creatinine)	55±18 ml/min/1.73m^2	35±15ml/min/1.73m^2	<0.001***
Proteinuria (g/day)	0.1±0.11	0.1±0.08	0.87
**Biopsy characteristics**			
Time after Ktx (days)	92.1±27.1	93.2±18.4	0.87

Values are expressed as % (n) or mean±SD. Student's t-test or Mann-Whitney test non-normally distributed variables was employed to obtain exploratory p-values. >90% of patients in both groups received basiliximab for induction, low dose steroids and mycophenolate. Loop diuretic dose is given as mg furosemide or equivalent.

Supplemental Table 4: Histologic characterization of allograft biopsies with and without loop diuretic exposure

	**No loop diuretic (n=21)**	**Loop diuretic (n=44)**	**p-value**
**Banff classification**			
Any rejection	24% (5)	25% (11)	1
Normal (Banff cat. 1)	0% (0)	0% (0)	1
Antibody mediated rej. (Banff cat. 2)	0% (0)	2% (1)	1
Cellular borderline rej. (Banff cat. 3)	14% (3)	14% (6)	1
T cell mediated rej. (Banff cat. 4)	9.5% (2)	8% (4)	1
BKV nephropathy	4.7% (1)	0% (0)	1
i (interstitial infiltrate in non-fibrotic cortex)	0.4±0.8	0.3±0.7	1
t (tubulitis)	0.4±0.8	0.5±0.9	0.46
v (arterial endotheliatis)	0±0	0.05±0.3	1
g (glomerulitis)	0±0	0.02±0.17	1
ptc (peritubular capillaritis)	0±0	0.05±0.32	1
C4d	0.5±1.1	0.02±0.16	0.028*
ci (cortical fibrosis)	0.6±0.6	0.8±0.7	0.4
ct (atrophic cortical tubuli)	1±0.3	1.1±0.4	0.26
cv (arterial stenosis)	0.05±0.2	0.2±1.4	1
cg (transplant glomerulopathy)	0±0	0±0	1
ti (interstitial infiltrate total cortex)	0.5±0.8	0.7±0.8	0.32
i-IFTA (interstitial fibrosis and tubular atrophy)	1.4±1.3	1.7±1.1	0.20
t-IFTA (interstitial fibrosis and tubular atrophy)	0.6±0.7	0.8±0.7	0.20
pvl (polyoma virus lesions)	0±0	0±0	1
ah (arterial hyalinosis)	0.6±0.7	0.8±0.7	0.19
mm (mesangial matrix expansion)	0.05±0.2	0.2±0.5	0.37
Banff cat. 6 type damage	71% (15)	68% (30)	1
Tubular damage	71% (15)	68% (30)	1
CNI toxicity	4.7% (1)	16% (7)	0.27

Values are expressed as % (n) or mean±SD. Mann-Whitney test was employed for exploratory p-values.

Supplemental Table 5: Compartmental CD8⁺ T cell densities and assessed areas in patients with and without histologic rejection

**CD8**	**No LD**		**LD**		p-value	
	Area (mm^2)	Nuclear density	Area (mm^2)	Nuclear density	area	Nuclear density
**All samples (n=65)**						
Cortex	0.34±0.2	0.84±2.5	0.35±0.2	2.8±4	0.563	0.0001***
Medulla	0.29±0.15	0.38±0.8	0.21±0.15	2.0±3.8	0.084	0.043*
**No rejection (n=49)**						
Cortex	0.27±0.17	0.87±2.8	0.11±0.44	2.9±4.7	0.14	0.0007***
Medulla	0.32±0.17	0.09±0.06	0.21±0.17	2.27±4.5	0.09	0.046*
**Any rejection (n=16)**						
Cortex	0.54±0.16	0.75±0.91	0.45±0.23	2.2±1.3	0.32	0.05
Medulla	0.22±0.11	1.0±1.4	0.19±0.09	1.4±1.7	0.66	0.66

"LD" indicates loop diuretic therapy. Mean±SD, Mann-Whitney tests for exploratory p-values.

Supplemental Table 6: Commonly up-regulated genes in NaCl and urea stimulated $CD8^+$ T cells

gene_id	gene_name	average ctrl	average NaCl	average urea	FC NaCl	p NaCl	FC urea	p urea
ENSG00000116731	PRDM2	228,770958	483,6162	458,856675	2,11397549	0,01304221	2,00574705	0,01026412
ENSG00000106443	PHF14	180,032286	391,93092	421,494984	2,17700352	0,0412999	2,34121887	0,00751299
ENSG00000061938	TNK2	78,0528125	225,192826	227,657847	2,88513403	0,00602622	2,91671549	0,00768127
ENSG00000129084	PSMA1	95,8126871	356,058121	246,821015	3,7161897	0,02859725	2,57607863	0,0240331
ENSG00000167325	RRM1	48,5490597	205,744164	385,759525	4,23786095	0,0429676	7,94576717	0,0072082
ENSG00000134294	SLC38A2	224,74655	961,319049	463,440062	4,2773473	0,02482109	2,0620564	0,03956366
ENSG00000148841	ITPRIP	14,1744585	62,9812456	153,819811	4,44329113	0,02022478	10,8519004	0,01621033
ENSG00000115317	HTRA2	9,89774558	45,5403828	93,7941392	4,60108642	0,02431749	9,47631342	0,00919743
ENSG00000069849	ATP1B3	107,445526	507,460952	375,586467	4,72296029	0,01708371	3,49559895	0,02055776
ENSG00000213983	AP1G2	16,9822021	100,804889	76,610747	5,93591386	0,01382321	4,5112375	0,02317554
ENSG00000247400	DNAJC3-DT	19,001528	114,995528	94,1907534	6,05190952	0,04343344	4,95700944	0,04172474
ENSG00000151466	SCLT1	20,3557237	135,033028	142,937159	6,63366383	0,00091895	7,02196402	0,03238349
ENSG00000181026	AEN	25,9968553	178,607969	204,202657	6,87036824	0,00545698	7,8548984	0,02614151
ENSG00000170234	PWWP2A	37,3358825	266,528926	262,096615	7,13868022	0,00237442	7,01996571	0,02449837
ENSG00000181555	SETD2	63,422181	459,43558	190,551041	7,24408358	0,0057221	3,00448578	0,03412054
ENSG00000253276	CCDC71L	5,278252	63,8089114	82,3160414	12,0890233	0,01530359	15,5953223	0,00086818
ENSG00000174839	DENND6A	1,00139731	53,0944698	84,1847993	53,0203839	0,04644623	84,0673312	0,01223202
ENSG00000102858	MGRN1	0,99761537	76,6551312	52,9162736	76,8383625	0,01258284	53,0427612	0,00386955
ENSG00000196754	S100A2	0,20027946	38,8095398	53,6032701	193,776933	0,04431073	267,642371	0,01532454
ENSG00000096696	DSP	0,20564093	60,5445654	20,4370401	294,418849	0,04587016	99,3821625	0,03048677

Supplemental Table 7: Commonly down-regulated genes in NaCl and urea stimulated CD8⁺ T cells

gene_id	gene_name	ave ctrl	ave nacl	ave urea	FC nacl	p nacl	fc urea	p urea
ENSG00000154134	ROBO3	121,435517	4,3411053	7,85582158	0,03574823	8,5529E-05	0,0646913	0,0001995
ENSG00000155660	PDIA4	228,028619	22,121732	83,0033583	0,09701296	0,00027173	0,36400413	0,01790465
ENSG00000182154	MRPL41	371,977874	143,06812	185,605199	0,3846146	0,00065378	0,49896839	0,02794397
ENSG00000140694	PARN	579,143053	181,25057	266,427379	0,31296338	0,00067836	0,46003725	0,02640504
ENSG00000100258	LMF2	149,300584	0	4,36434532	0	0,00093258	0,02923194	0,00117988
ENSG00000141506	PIK3R5	260,792936	50,2358228	124,294367	0,19262724	0,00147839	0,47660174	0,02152584
ENSG00000088766	CRLS1	87,7897428	12,7866117	32,0705095	0,14565041	0,0018092	0,36531044	0,04590753
ENSG00000118972	FGF23	52,329315	22,9794267	24,8597745	0,43913104	0,00190411	0,47506402	0,00506719
ENSG00000165591	FAAH2	107,529385	13,0233159	0	0,12111402	0,00200936	0	0,0001459
ENSG00000139193	CD27	416,252457	169,066021	174,65056	0,40616222	0,00328885	0,41957845	0,03326989
ENSG00000132478	UNK	129,227576	8,15067323	27,5342502	0,06307224	0,00344941	0,21306792	0,03515166
ENSG00000213619	NDUFS3	372,098784	177,965739	155,921898	0,47827552	0,00440553	0,41903361	0,00901272
ENSG00000139946	PELI2	65,4338228	1,59173861	13,4467984	0,02432593	0,00453686	0,20550226	0,01939437
ENSG00000184384	MAML2	300,408584	85,2449007	114,091773	0,2837632	0,00458689	0,37978866	0,03440722
ENSG00000134905	CARS2	158,907733	10,8428122	49,8969489	0,06823338	0,00547621	0,3139995	0,02689644
ENSG00000131269	ABCB7	143,563571	19,2765199	50,6979546	0,13427167	0,00603154	0,35313941	0,03578281
ENSG00000133106	EPSTI1	1150,70886	552,333455	290,557797	0,47999409	0,00692409	0,25250331	0,00062848
ENSG00000151136	BTBD11	162,474411	94,9841988	51,2207355	0,58461021	0,00788463	0,31525417	0,02235092
ENSG00000099899	TRMT2A	190,202131	27,7783043	12,7697276	0,14604623	0,00824233	0,06713767	0,00471173
ENSG00000064490	RFXANK	275,05538	43,9146356	39,681674	0,15965743	0,00932798	0,14426794	0,00699375
ENSG00000157916	RER1	398,119848	192,47981	175,016106	0,48347203	0,01167829	0,43960658	0,0169765
ENSG00000183751	TBL3	90,4091695	17,9762693	34,1271911	0,19883237	0,01178078	0,37747489	0,04162642
ENSG00000120875	DUSP4	209,874289	24,6030937	9,32611699	0,11722776	0,01240733	0,04443668	0,00687208
ENSG00000111481	COPZ1	357,397052	140,835732	87,2291388	0,39405958	0,01344315	0,24406787	0,00656805
ENSG00000198931	APRT	589,593449	342,471946	237,800295	0,58086118	0,01390169	0,40332927	0,02085269
ENSG00000198646	NCOA6	182,072571	54,5522654	57,1548136	0,29961825	0,01411414	0,31391227	0,00811549
ENSG00000079616	KIF22	206,377925	75,0898821	43,267647	0,36384648	0,01508284	0,20965249	0,01309665
ENSG00000010626	LRRC23	61,0594127	0	0	0	0,01575577	0	0,01575577
ENSG00000138629	UBL7	229,554369	72,0072778	46,9157671	0,31368289	0,01685894	0,20437758	0,0082534

ENSG00000122641	INHBA	556,254188	171,178228	222,580157	0,30773382	0,01758015	0,40014109	0,01859276
ENSG00000139629	GALNT6	189,622328	26,332544	43,5244582	0,13886837	0,01819785	0,22953235	0,02824244
ENSG00000234663	LINC01934	256,522135	61,4358085	74,1082588	0,23949516	0,01974883	0,28889616	0,00932596
ENSG00000167550	RHEBL1	51,7144787	7,96510415	0	0,15402078	0,0210976	0	0,0043738
ENSG00000089327	FXYD5	423,541897	185,702947	190,063978	0,43845236	0,02291935	0,44874894	0,03380467
ENSG00000119777	TMEM214	164,038013	61,9057325	10,2143186	0,3773865	0,02323601	0,062268	0,00080537
ENSG00000136937	NCBP1	157,764589	20,4632075	20,7662067	0,12970723	0,02354938	0,13162781	0,02413085
ENSG00000105287	PRKD2	389,14391	207,377701	149,528859	0,53290748	0,0237207	0,3842508	0,00353778
ENSG00000106178	CCL24	171,080368	2,31525616	0	0,01353315	0,02389884	0	0,02258917
ENSG00000260032	NORAD	602,729154	233,284288	246,426497	0,38704663	0,02447802	0,40885113	0,01986862
ENSG00000204899	MZT1	86,1809023	24,9457777	15,0994275	0,2894583	0,02554405	0,17520619	0,015011
ENSG00000257119	EEF1B2P4	0,57218962	0	0	0	0,02557676	0	0,02557676
ENSG00000276168	RN7SL1	442,26816	89,5122576	122,540268	0,20239363	0,02629894	0,27707233	0,04125
ENSG00000149923	PPP4C	264,870311	103,062383	103,342433	0,38910508	0,02729211	0,39016239	0,0417688
ENSG00000119285	HEATR1	242,322441	123,402423	81,5119043	0,50924884	0,02965932	0,33637786	0,03099973
ENSG00000129484	PARP2	63,4042115	0	0	0	0,02967919	0	0,02967919
ENSG00000145996	CDKAL1	265,897237	84,6000922	113,958126	0,31816838	0,03029757	0,42857958	0,04740999
ENSG00000101246	ARFRP1	88,6796355	120,242289	1,45478177	1,35591772	0,03054057	0,01640491	5,868E-05
ENSG00000174780	SRP72	637,077334	423,064158	306,412892	0,66407033	0,03214964	0,48096656	0,00083982
ENSG00000197020	ZNF100	23,3634661	0	0	0	0,03368603	0	0,03368603
ENSG00000156050	FAM161B	38,4965394	0	0,58191271	0	0,03596311	0,01511597	0,0380802
ENSG00000172183	ISG20	1408,13542	942,360103	490,162798	0,66922548	0,03967294	0,34809351	0,00094244
ENSG00000089639	GMIP	285,878343	117,529302	106,001341	0,41111649	0,04112767	0,37079179	0,0489591
ENSG00000042286	AIFM2	44,4655606	0	0	0	0,04227435	0	0,04227435
ENSG00000159496	RGL4	105,542189	23,19408	15,5398885	0,21976122	0,0459065	0,14723864	0,02363417
ENSG00000090104	RGS1	283,675981	79,3925329	37,0005518	0,27987048	0,04687639	0,13043244	0,02304922
ENSG00000267508	ZNF285	53,049637	0	0	0	0,04808685	0	0,04808685

Supplemental Table 8: Fold change of gene expression in parallely regulated genes in naïve and total NaCl-stimulated CD8⁺ T cells

	Present dataset		Scirgolea et al., NI 2024	Soll et al., NI 2024
	+ NaCl	+ Urea		
**PRRG4**	337,901087	287,577694	10,6642334	13,2645057
**DBNDD2**	172,988444	0	2,41606905	2,48922358
**SLC39A8**	94,1702244	77,5988557	1,91977885	2,08940479
**SFMBT2**	60,2946743	3,87034139	1,91017168	2,15037951
**PRKAG2-AS1**	55,6120133	14,1912518	5,01874405	6,20530867
**POLR3H**	11,6193702	4,12424589	2,08744982	1,76045016
**AKAP1**	11,1750188	2,56137882	1,9213477	2,10592686
**ASRGL1**	9,74620903	3,63372511		2,52446258
**SLC5A3**	7,50885861	0,80153325	3,80782553	4,17571224
**CDK2AP1**	6,86711778	2,60353674	4,3340837	4,37069266
**PGP**	5,43868172	1,7791412	2,01430044	2,12717662
**ATP1B3**	4,72296029	3,49559895	2,2349978	2,44412373
**CTPS1**	4,48172469	1,89497012	2,33193403	2,50116254
**RBFA**	4,30715139	6,09829002	2,16365987	2,23666201
**MSMO1**	3,52038934	1,73479132	2,10970773	2,3630765
**MRPS6**	3,04327773	0,47642416	3,4101951	3,78668743
**PRMT1**	2,88189047	0,24736862	1,96641651	2,12451048
**JAK3**	0,48123036	0,69635457	0,38160523	0,43048401
**CD27**	0,40616222	0,41957845	0,41077339	0,51733069
**NUAK2**	0,38500391	1,46560237	0,15126972	0,17068591
**KDM7A**	0,32656271	1,00667795	0,39577776	0,41724795
**TTC39B**	0,3080859	0,59181478	0,48727932	0,50847458
**LMBR1L**	0,00947101	1,53056758	0,44258554	0,49070632
**MEGF6**	0	0,81012845	0,15315614	0,1766427

Supplemental references:

1. Naesens M, et al. The Banff 2022 Kidney Meeting Report: Reappraisal of microvascular inflammation and the role of biopsy-based transcript diagnostics. *Am J Transplant*. 2024;24(3):338–349.
2. Bräsen JH, et al. Macrophage density in early surveillance biopsies predicts future renal transplant function. *Kidney Int*. 2017;92(2):479–489.
3. Apaolaza PS, Petropoulou P-I, Rodriguez-Calvo T. Whole-Slide Image Analysis of Human Pancreas Samples to Elucidate the Immunopathogenesis of Type 1 Diabetes Using the QuPath Software. *Front Mol Biosci*. 2021;8:689799.
4. Bankhead P, et al. QuPath: Open source software for digital pathology image analysis. *Sci Rep*. 2017;7(1):16878.
5. Goldspink A, et al. Kidney medullary sodium chloride concentrations induce neutrophil and monocyte extracellular DNA traps that defend against pyelonephritis in vivo. *Kidney International*. 2023;104(2):279–292.
6. Hüsing AM, et al. Myeloid CCR2 Promotes Atherosclerosis after AKI. *J Am Soc Nephrol*. 2022;ASN.2022010048.
7. Ewels PA, et al. The nf-core framework for community-curated bioinformatics pipelines. *Nat Biotechnol*. 2020;38(3):276–278.
8. The Bioconda Team, et al. Bioconda: sustainable and comprehensive software distribution for the life sciences. *Nat Methods*. 2018;15(7):475–476.
9. Da Veiga Leprevost F, et al. BioContainers: an open-source and community-driven framework for software standardization. *Bioinformatics*. 2017;33(16):2580–2582.
10. Di Tommaso P, et al. Nextflow enables reproducible computational workflows. *Nat Biotechnol*. 2017;35(4):316–319.
11. Xu K, et al. Unique Transcriptional Programs Identify Subtypes of AKI. *JASN*. 2017;28(6):1729–1740.
12. Scirgolea C, et al. NaCl enhances CD8+ T cell effector functions in cancer immunotherapy. *Nat Immunol*. 2024;25(10):1845–1857.
13. Soll D, et al. Sodium chloride in the tumor microenvironment enhances T cell metabolic fitness and cytotoxicity. *Nat Immunol*. 2024;25(10):1830–1844.
14. Yokose T, et al. Dysfunction of infiltrating cytotoxic CD8+ T cells within the graft promotes murine kidney allotransplant tolerance. *J Clin Invest*. 2024;134(16):e179709.
